# Variations in dysfunction of sister chromatid cohesion in *esco2* mutant zebrafish reflect the phenotypic diversity of Roberts syndrome

**DOI:** 10.1242/dmm.019059

**Published:** 2015-08-01

**Authors:** Stefanie M. Percival, Holly R. Thomas, Adam Amsterdam, Andrew J. Carroll, Jacqueline A. Lees, H. Joseph Yost, John M. Parant

**Affiliations:** 1 Department of Pharmacology and Toxicology, University of Alabama at Birmingham, Birmingham, AL 35294, USA; 2 David H. Koch Institute for Integrative Cancer Research at MIT, Massachusetts Institute of Technology, Cambridge, MA 02139, USA; 3 Department of Clinical and Diagnostic Science, University of Alabama at Birmingham, Birmingham, AL 35294, USA; 4 Department of Neurobiology and Anatomy, University of Utah, Salt Lake City, UT 84132, USA

**Keywords:** *esco2*, Sister chromatid cohesion, *In vivo* imaging, Zebrafish, p53, Genomic instability, Aneuploidy

## Abstract

Mutations in ESCO2, one of two establishment of cohesion factors necessary for proper sister chromatid cohesion (SCC), cause a spectrum of developmental defects in the autosomal-recessive disorder Roberts syndrome (RBS), warranting *in vivo* analysis of the consequence of cohesion dysfunction. Through a genetic screen in zebrafish targeting embryonic-lethal mutants that have increased genomic instability, we have identified an e*sco2* mutant zebrafish. Utilizing the natural transparency of zebrafish embryos, we have developed a novel technique to observe chromosome dynamics within a single cell during mitosis in a live vertebrate embryo. Within *esco2* mutant embryos, we observed premature chromatid separation, a unique chromosome scattering, prolonged mitotic delay, and genomic instability in the form of anaphase bridges and micronuclei formation. Cytogenetic studies indicated complete chromatid separation and high levels of aneuploidy within mutant embryos. Amongst aneuploid spreads, we predominantly observed decreases in chromosome number, suggesting that either cells with micronuclei or micronuclei themselves are eliminated. We also demonstrated that the genomic instability leads to p53-dependent neural tube apoptosis. Surprisingly, although many cells required Esco2 to establish cohesion, 10-20% of cells had only weakened cohesion in the absence of Esco2, suggesting that compensatory cohesion mechanisms exist in these cells that undergo a normal mitotic division. These studies provide a unique *in vivo* vertebrate view of the mitotic defects and consequences of cohesion establishment loss, and they provide a compensation-based model to explain the RBS phenotypes.

## Abstract (precis)

**Summary:** *In vivo* analysis of zebrafish *esco2* mutants reveals extensive genomic instability and activation of DNA-damage-response pathways, although some cells have compensatory cohesion and divide normally.

## INTRODUCTION

Sister chromatid cohesion (SCC) is a dynamic, cell-cycle-dependent process that is essential for proper segregation of chromosomes. A protein complex forms a cohesin ring comprised of SMC1a, SMC3, RAD21 and STAG1/2 that is associated, or ‘loaded’, onto DNA during the G1 phase of the cell cycle by the NIPBL and Mau-2 proteins (Losada, 2008; Nasmyth and Haering, 2009; Ocampo-Hafalla and Uhlmann, 2011). Upon entry into S-phase, as the sister chromatids are being synthesized, the two establishment of cohesion factors, ESCO1 and ESCO2, establish cohesion by securing the cohesin ring around sister chromatids (Skibbens et al., 1999; Hartman et al., 2000; Homer et al., 2005; Skibbens, 2009; Terret et al., 2009). During mitosis, cohesion is removed in two steps: (1) during the transition from prophase to prometaphase, cohesion between chromatid arms is removed through the anti-establishment pathway involving WAPAL, whereas the centromeric cohesion is protected by establishment and/or maintenance factors, including SGO1, SGO2 and sororin (Salic et al., 2004; Rankin et al., 2005; Gandhi et al., 2006; Kueng et al., 2006; Sutani et al., 2009); and (2) upon proper bipolar attachment of all chromosomes, the cell will undergo metaphase-to-anaphase transition in which the enzyme separase cleaves the remaining centromeric cohesin rings, allowing sister chromatid segregation to opposing spindle poles (Waizenegger et al., 2000; Sonoda et al., 2001; Losada, 2008). Improper attachments or lack of kinetochore tension results in maintenance of the spindle assembly checkpoint (SAC), preventing the metaphase-to-anaphase transition (Li and Nicklas, 1995; Nicklas et al., 1995). Beyond the mitotic function of SCC, studies have expanded its function to a wide assortment of cellular functions, including DNA repair (Sjögren and Nasmyth, 2001; Kim et al., 2002; Schar et al., 2004; Strom and Sjogren, 2005; Gause et al., 2008; Göndör and Ohlsson, 2008; Heidinger-Pauli et al., 2009; Covo et al., 2010; Lu et al., 2010; Dorsett and Ström, 2012), gene regulation (Horsfield et al., 2007, 2012; Pauli et al., 2010; Dorsett and Ström, 2012), ribogenesis (Xu et al., 2013) and centrosome duplication (Schöckel et al., 2011; Yamada et al., 2012).

Traditionally, SCC is studied in the context of individual cells. However, the recent discovery that components of SCC are responsible for human developmental disorders and tumorigenesis has illuminated the requirement for *in vivo* analysis (Duijf and Benezra, 2013; Liu and Krantz, 2008). Roberts syndrome (RBS) is caused by recessive mutations exclusively in ESCO2. Severity of disease varies between affected individuals, from prenatal lethal to viable beyond 30 years of age, and there is also a variety of specific developmental phenotypes, including microcephaly, craniofacial defects, mental retardation, limb deformities and growth retardation (Schüle et al., 2005; Vega et al., 2010). In addition to these hallmark phenotypes, some individuals with RBS also display cardiac defects and corneal opacity, as well as other less prominent phenotypes (Vega et al., 2010). Interestingly, among the few individuals that live beyond 30, some develop tumors at an early age, suggesting a cancer predisposition (Wenger et al., 1988; Ogilvy et al., 1993; Schüle et al., 2005). Metaphase spreads from RBS patients display centromeric separation and low levels of aneuploidy, suggesting that the defects are associated with SCC and mis-segregation of chromosomes (German, 1979; Tomkins et al., 1979). How such an essential gene could have developmental phenotypes is unclear. There are two vertebrate paralogs (ESCO1 and ESCO2) of the yeast ECO1 required for establishing SCC (Skibbens et al., 1999; Toth et al., 1999). Both ESCO1 and ESCO2 contain acetyl-transferase domains and have the ability to acetylate SMC3, locking it in the cohesion position. Although they seem to have overlapping activities, the Zou lab demonstrated that they have non-redundant functions because both must be depleted to have complete SCC loss in HeLa cells (Hou and Zou, 2005). Potentially, the tissue-specific phenotypes (e.g. limb, craniofacial, neural, etc.) found in individuals with RBS (exclusively due to ESCO2 mutations) are associated with differential requirements for ESCO1 or ESCO2 in different tissues. In addition to RBS, there are other developmental syndromes caused by mutations in SCC components: (1) Cornelia de Lange syndrome (CdLS), caused by mutations in NIPBL, SMC1, SMC3, HDAC8 and RAD21 (Krantz et al., 2004; Tonkin et al., 2004; Musio et al., 2006; Deardorff et al., 2007; Liu and Krantz, 2008); (2) Warsaw breakage syndrome (WABS), caused by mutations in DDX11 (van der Lelij et al., 2010; Capo-Chichi et al., 2013); and (3) chronic atrial and intestinal dysrhythmia (CAID), caused by mutations in SGOL1 (Chetaille et al., 2014). Importantly, with the exception of CdLS, these syndromes all display premature chromatid separation in metaphase spreads, suggesting that this process is pathogenic in the diseases.

**TRANSLATIONAL IMPACT**

**Clinical issue**

Defects in sister chromatid cohesion (SCC; the process in which sister chromatids are paired during the cell cycle) can lead to multiple human disorders, including but not limited to infertility, birth defects and cancer. Although we know much about SCC in the context of individual cells, the identification of Roberts syndrome (RBS), a developmental disorder caused by mutations in the cohesion establishment gene *ESCO2*, implies that further *in vivo* studies on SCC are required to understand the disease pathology. Moreover, the unique variety of phenotypes and wide range of severity of the disease suggest that there are differences in cellular response and potential complex genetic interactions that are presently not understood.

**Results**

This study describes the first characterization of an *esco2* mutant zebrafish and develops the use of fluorescence single-cell time-lapse confocal imaging to monitor the dynamics of chromosome movements during mitosis in live zebrafish embryos. Loss of Esco2 results in embryonic lethality due to extensive chromosome scattering upon entrance into prometaphase, in a prolonged mitotic delay and, ultimately, in imprecise chromosome segregation upon division. Various forms of genomic instability result from these divisions, including the development of micronuclei and anaphase bridges, which activate a neural-tube-specific p53-dependent apoptotic response in early development. Most noteworthy is that some cells divide with normal mitotic progression and lack genomic instability in the *esco2* mutant, which suggests that compensatory cohesion establishment mechanisms are in place to allow for normal mitotic progression and division in these cells.

**Implications and future directions**

This work exemplifies the novelty of observing the dynamics of cell divisions in a live vertebrate organism. It also suggests, for the first time, that compensatory mechanisms might influence the spectrum of phenotypes observed in RBS. Further studies will investigate tissue-specific differences, identify the compensatory mechanisms and visualize the dynamics that regulate such mechanisms. Globally, this research will impact not only developmental disorders but also infertility and tumorigenesis. Somatic mutations have been found in a multitude of cancer types, and key defects in SCC have been associated with increased tumorigenesis. Female, and most recently male, infertility has also been shown to be caused by defects in SCC, further supporting the importance of understanding how SCC is regulated. By identifying key compensatory mechanisms, these could be exploited as therapeutic targets for treating SCC-associated human diseases.

In the wake of identifying the causal gene for RBS, a few animal models provided key initial studies for the role of ESCO2 in RBS. The mouse knockout of *Esco2* is eight-cell-stage lethal, which unfortunately limits *in vivo* multicellular analysis (Whelan et al., 2012). Utilizing a conditional allele, *Esco2*-null mouse embryonic fibroblasts were generated, and cell culture analysis revealed partial chromatid separation and genomic instability. A zebrafish morphant (morpholino-derived partial knockdown) study focusing largely on RBS phenotypes indicated that zebrafish can recapitulate many of the morphological RBS-like phenotypes (Mönnich et al., 2011). Importantly, they observed that different RBS phenotypes occurred at varying levels of morpholino knockdown, suggesting that the degree of SCC loss correlates with the severity of RBS phenotypes. Notably, they describe that the mitotic and apoptotic phenotypes associated with induction of stress genes, but not cohesin-dependent gene expression changes, drive the RBS phenotypes. Although these studies focus on key RBS phenotypes, little knowledge is known about the *in vivo*, cellular events that result from Esco2 loss.

In this study, we characterize the dynamic, *in vivo*, cellular consequences that present in an *esco2* mutant zebrafish. We find that, in addition to prolonged mitotic arrest, there are chromosome segregation defects, micronuclei formation and genomic instability. In addition, we demonstrate a cohesion compensatory mechanism in a portion of cells of an *esco2* mutant embryo, supporting the idea that different cells, and potentially tissues, have different sensitivities (or redundancies) to Esco2 loss.

## RESULTS

### *esco2* retroviral insertion mutant identified through a unique p53 genetic screen

**Fig. 1.**
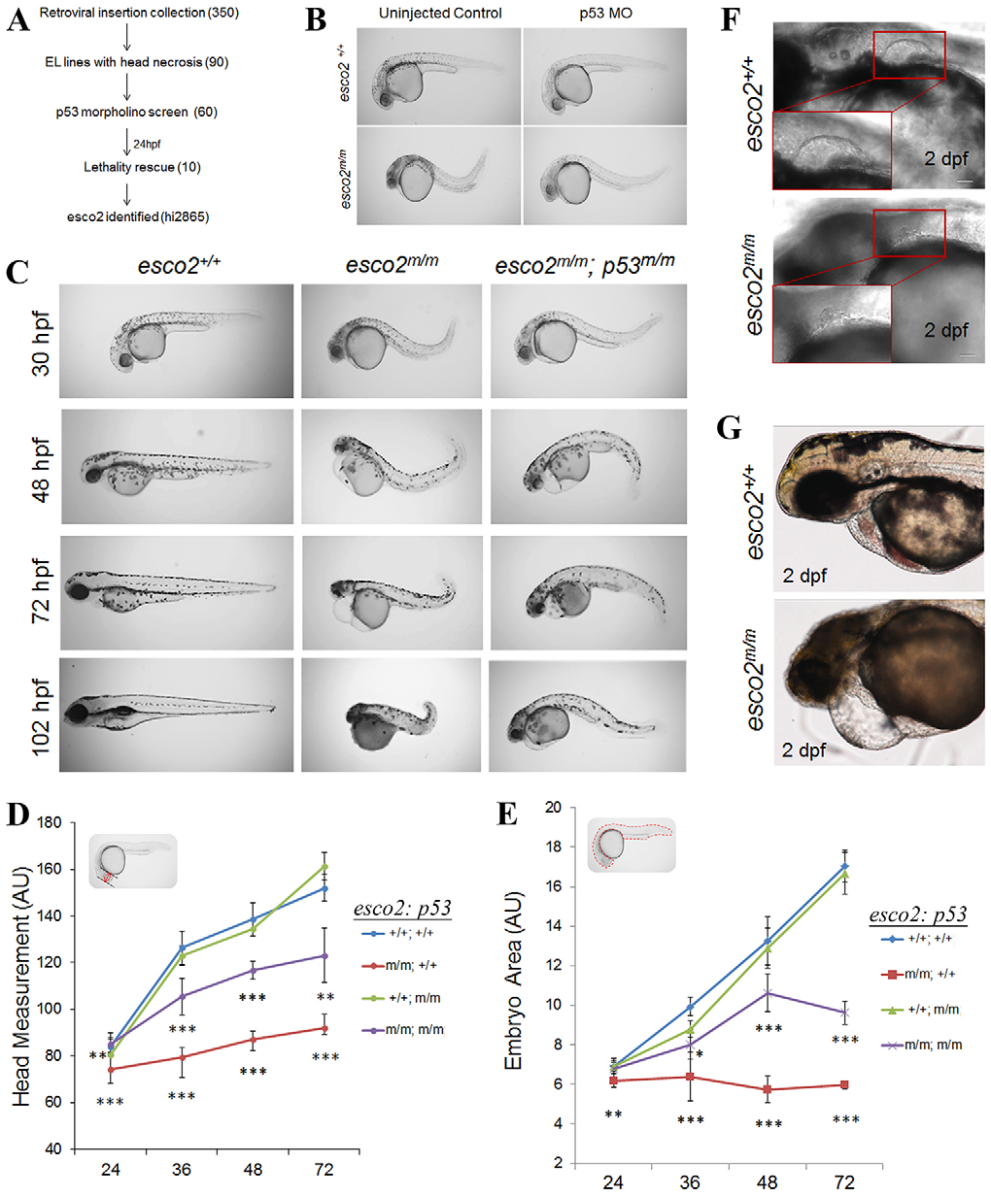
**Genetic screen identifies the retroviral insertion embryonic lethal mutant, hi2865.** (A) Design of the screen used to identify insertion mutants exhibiting microcephaly and head necrosis, amongst 350 embryonic lethal (EL) mutants, that are rescued by morpholino (MO) knockdown of *p53* (numbers in parentheses indicate the number of mutants at each stage) at 24 hpf. (B) Brightfield images of a 24-hpf hi2865 sibling and homozygous mutant (henceforth *esco2*^*hi2865/hi2865*^ will be referred to as *esco2*^*m/m*^) embryos injected or uninjected with *p53* MO. (C) *esco2^+/+^*, *esco2^m/m^* and *esco2^m/m^; p53^dzy7/dzy7^* (henceforth *p53^dzy7/dzy7^* will be referred to as *p53*^*m/m*^) gross morphological phenotypes between 30 hpf and 102 hpf (4 dpf). (D) Head measurements and (E) embryo area of *esco2^+/+^*, *esco2^m/m^*, *esco2^+/+^; p53^−/−^* and *esco2^m/m^; p53^−/−^* were measured using ImageJ in arbitrary units (*n*=5/genotype, mean±s.d., *P<0.05, **P<0.01, ***P<0.001; significance values shown below the red line are derived from comparing *esco2*^*m/m*^ versus *esco2*^+/+^; significance values shown below the purple line are from comparing *esco2*^*m/m*^*; p53*^*m/m*^ versus *esco2^m/m^*). Insets in each graph depict the measurement parameters highlighted in red. (F,G) DIC images depict fin and heart defects, respectively, in *esco2^m/m^*. Insets detail normal fin and remnant fin bud. Scale bars: 50 µm.

During tumorigenesis, p53 is activated following cellular stress, resulting in sequestration or termination of the stressed cell (Junttila and Evan, 2009; Meek, 2009). Through our previous studies, we observed that, either following loss of *mdm2*, the negative regulator of p53, or following ionizing radiation (IR) treatment, zebrafish embryos displayed a darkening of the head region, referred to as head necrosis at 24 hpf (hours post-fertilization), which results from an increase in apoptosis in the neural tube (Berghmans et al., 2005; Sidi et al., 2008; Parant et al., 2010; Toruno et al., 2014). To better understand (1) which cellular stresses activate p53, (2) what birth defects result from p53 activation and (3) the mechanisms of p53 activation following the cellular stress, we devised a genetic screen to identify p53-dependent embryonic-lethal zebrafish mutants. More specifically, this screen was designed to identify embryonic-lethal mutants with head necrosis that can be fully or partially rescued by loss of p53. We utilized the Hopkins retroviral insertion-derived embryonic-lethal mutant collection as a source of embryonic-lethal mutants (Fig. 1A) (Amsterdam et al., 2004). From this collection, we selected 90 embryonic-lethal mutants with the head necrosis phenotype. To determine whether the head necrosis is p53-dependent, we injected half of the clutch from 60 heterozygous intercrossed mutant families with a p53-knockdown morpholino. Injected and uninjected embryos were monitored for head necrosis and other morphological phenotypes between 20 and 48 hpf. From this primary screen, we identified ten mutants that displayed partial rescue following *p53* morpholino injection (hi821a, hi1045, hi1477, hi2404, hi2877b, hi2865, hi2975, hi3635, hi3662, hi3820a). None of the ten mutants displayed complete rescue following *p53* morpholino injections, suggesting either that additional non-p53-dependent defects are present or that the morpholino knockdown was not complete or was no longer effective at inhibiting p53 activity over time.

One of the mutants identified was the hi2865 line, with a retroviral insertion in intron 1 of the ENDARG00000014685 gene (Fig. 1B; supplementary material Fig. S1A). To determine whether homozygosity for this insertion correlated with the mutant phenotype observed, we performed high-resolution melt curve analysis (HRMA), in addition to multiplex allele-specific PCR (supplementary material Fig. S1B and C, respectively). The results confirmed that 100% (*n*=11 of 11) of mutant embryos were homozygous for the insertion, and that none (*n*=32 of 32) of the normal siblings were homozygous for the insertion. ENDARG00000014685 has homology to the establishment of cohesion homolog 2 gene (*ESCO2*), and displays strong synteny with the mouse and human genomic region surrounding *ESCO2* (supplementary material Fig. S1D).

Previous work has shown that the majority of intron 1 retroviral insertions knock down the endogenous gene transcript by greater than 80% (Wang et al., 2007). We performed qRT-PCR on pooled wild-type (WT) and mutant embryos (henceforth *esco2*^*hi2865/hi2865*^ will be referred to as *esco2*^*m/m*^) at 18 hpf, 30 hpf and 48 hpf. By 18 hpf, we observed ∼7% of normal transcript and by 48 hpf ∼2% of transcript (supplementary material Fig. S2), indicating: (1) the retroviral insertion diminished the *esco2* transcript by >95%, and (2) by 18 hpf, the majority of maternal transcript is absent. To further validate that these morphological phenotypes are due to disruption of *esco2*, we have recently generated an *esco2* exon 3 frameshift mutation (*esco2*^+13^), using the CRISPR genome-editing system (Hwang et al., 2013; Jao et al., 2013; Thomas et al., 2014). Homozygous *esco2*^+13/+13^ embryos have the same gross morphological phenotypes (supplementary material Fig. S3A,B) and decrease in *esco2* mRNA expression (supplementary material Fig. S2) as *esco2^m/m^* embryos. Note that the reduced mRNA expression in the *esco2*^+13/+13^ embryos is most likely due to nonsense-mediated decay due to the premature stop in the +13 transcript.

### *esco2* deficiency has many severe RBS-like phenotypes

We wanted to determine whether our *esco2* mutant animal modeled the human RBS patient phenotypes. Although there are strong variations in the RBS phenotypes, individuals with prenatal-lethal RBS consistently have microcephaly, growth retardation, craniofacial defects and limb deformities (Schüle et al., 2005). Gross morphology at 30 hpf of the *esco2^m/m^* (Amsterdam et al., 2004) zebrafish showed head necrosis and growth retardation (Fig. 1C). At 48 hpf, the head size was reduced dramatically compared to the WT. Importantly, head and gross embryo size measurements suggest a lack of growth compared to WT (Fig. 1D,E), reminiscent of human microcephaly and growth retardation. Furthermore, in WT embryos at 48 hpf, the early embryonic pectoral fin (analogous to the forelimb in mammals) had formed; however, the fin was absent in the majority of mutants (seven out of ten), and only a small nub in the other mutant embryos (Fig. 1F), indicating defects in limb formation. Unfortunately, by 4 days post-fertilization (dpf) all *esco2*^*m/m*^ embryos (*n*=20) were almost completely degraded (Fig. 1C), obscuring the ability to address craniofacial abnormalities because craniofacial bone/cartilage does not begin to appear until 5 dpf. Although not a hallmark of RBS, heart defects are prevalent in 25-75% of patients (Vega et al., 2010). We observed that, although the majority of *esco2* mutant embryos seem to undergo proper morphogenesis (formation of an atrium and ventricle) and have 1:1 atrioventricular (A-V) contractions, they do not undergo proper heart looping and often have variable heartbeat rates and lack of blood flow (Fig. 1G; supplementary material Movies 1 and 2). Whereas the phenotype of RBS patients is quite pleiotropic (presumed to be due to genetic diversity), the zebrafish phenotypes are very consistent and most likely reflect nearly isogenic backgrounds of our zebrafish.

### p53 activation and neural tube apoptosis is an early consequence of loss of Esco2

**Fig. 2.**
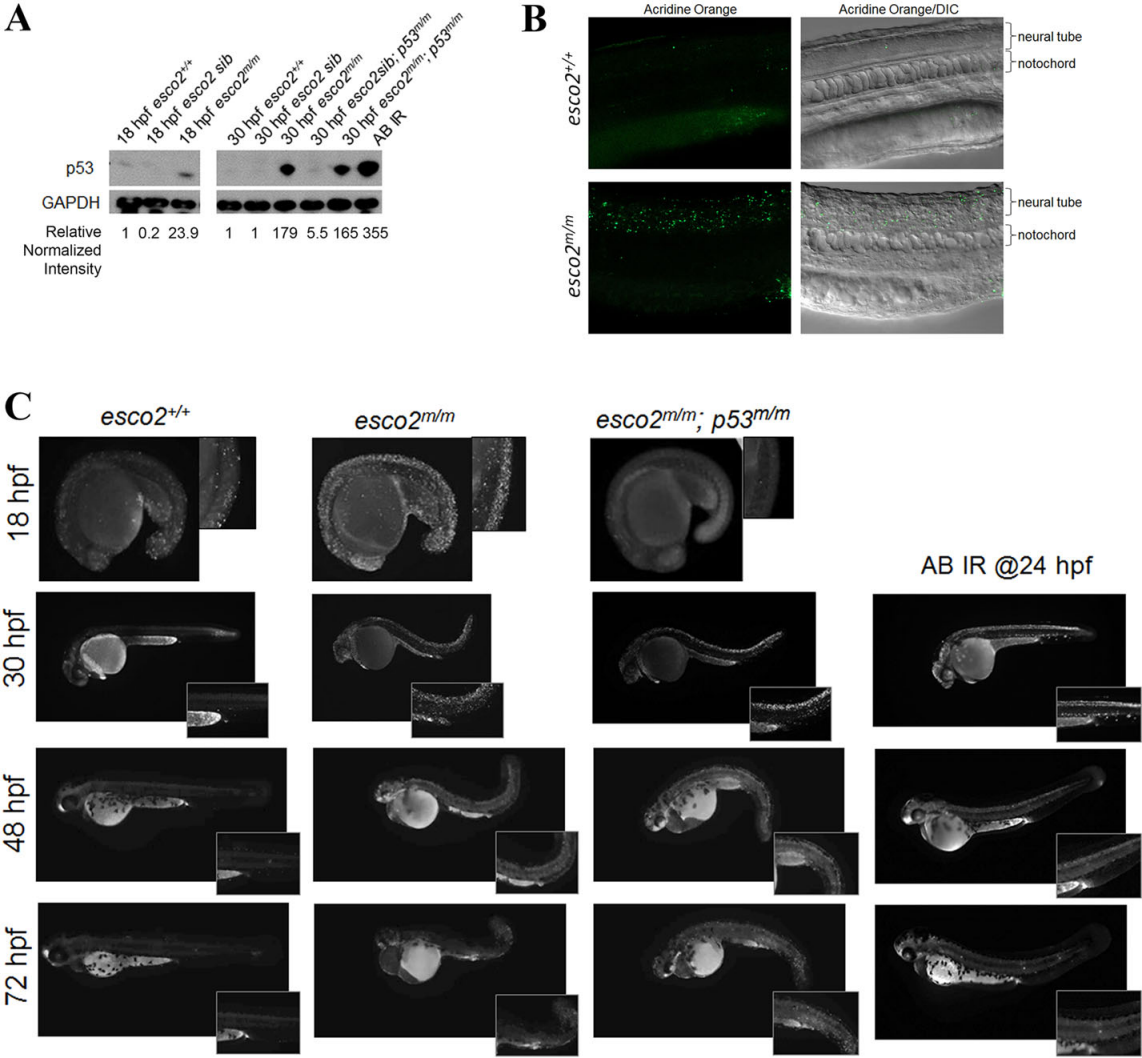
**p53 activation and neural tube apoptosis is an early consequence of loss of *esco2*.** (A) Western blot for p53 protein levels in protein extracts from AB (*esco2*^+/+^), *esco2* sib (*esco2*^+/+^ and *esco2*^*+/m*^), *esco2* mutant (*esco2*^*m/m*^), *esco2*sib; *p53*^*m/m*^ and *esco2*^*m/m*^*; p53*^*m/m*^ embryos at 18 and 30 hpf. Irradiated (IR) embryos at 100 Gy were used as a positive control. Relative intensities were determined using ImageJ; each sample was normalized to GAPDH intensity, and then relative expression was calculated against *esco2*^+/+^ (relative normalized intensity=1). (B) Fluorescent and DIC/fluorescent merge images of *esco2^+/+^* and *esco2^m/m^* 24-hpf embryos stained with acridine orange. (C) Acridine orange time-course staining spatially displaying apoptotic cells in *esco2^+/+^*, *esco2^m/m^* and *esco2^m/m^; p53^m/m^*. AB embryos irradiated at 24 hpf were used as a positive control for DNA-damage-induced neural tube apoptosis. Insets depict higher magnification to visualize neural tube apoptosis.

Initially, we identified this mutant by the ability of a *p53* morpholino to partially rescue the gross morphological phenotypes. To biochemically confirm p53 activation, we probed protein extracts from controls (AB strain), *esco2* mutant and *esco2* sibling embryos for p53. We observed that p53 stabilization was strong in *esco2^m/m^* embryos (179-fold at 30 hpf) but not in WT controls (Fig. 2A). To determine whether genetic loss of p53 could rescue our *esco2^m/m^* phenotypes, we generated *esco2^hi2865/hi2865^; p53^dzy7/dzy7^* mutant embryos (henceforth referred to as *esco2^m/m^;* *p53^m/m^*), in which the *dzy7* allele has an I166T mutation in the DNA-binding domain of p53, rendering it transcriptionally inactive (Parant et al., 2010). In contrast to the prominent head necrosis phenotype observed in *esco2^m/m^* alone, *esco2^m/m^; p53^m/m^* showed severely diminished head necrosis (Fig. 1C) with stabilization of the p53 mutant protein (Fig. 2A). As observed by gross morphology, our quantitative measurements of microcephaly and growth retardation indicate a partial rescue of these phenotypes (Fig. 1D,E).

One of the major outcomes of p53 activation is apoptosis. Therefore, to further understand the *esco2* mutant phenotype, we stained *esco2^+/+^* and *esco2^m/m^* embryos for apoptosis at 18, 24, 30, 48 and 72 hpf using the live apoptotic dye acridine orange. Interestingly, we observed that, at 18, 24 and 30 hpf, *esco2^m/m^* embryos had increased levels of apoptosis with a higher proportion of apoptosis predominantly in the neural tube at 24 and 30 hpf (Fig. 2B,C). Interestingly, this increase in neural tube apoptosis is consistent with our previously described increase in neural tube apoptosis following IR treatment (Fig. 2C). By 48 hpf and later, apoptosis was no longer observed in the mutant embryos, suggesting that the sensitivity to stress-inducing apoptosis or the stress no longer present in these cells, or that all of the cells sensitive to the stress have died. At 18 hpf in the p53 mutant background, we observed an absence of apoptosis in *esco2^m/m^; p53^m/m^* embryos; however, by 30 hpf we observed a similar level of apoptosis within the *esco2^m/m^; p53^m/m^* embryos as the *esco2*^*m/m*^ embryos (Fig. 2C). This suggests that the initial consequence of Esco2 loss results in p53-induced apoptosis. However, a subsequent stress-induced apoptosis occurs in the absence of p53. This is also reminiscent of irradiation experiments in the p53 mutant embryo, where this initial apoptosis following IR treatment is abrogated in a p53 mutant background but a secondary apoptosis occurs later (Parant et al., 2010). Our interpretation is that, although p53 loss abrogates the response, the damage or stress is still present and detrimental to the cell.

### Esco2 deficiency results in embryonic lethality associated with chromosome scattering and increased mitotic index

**Fig. 3.**
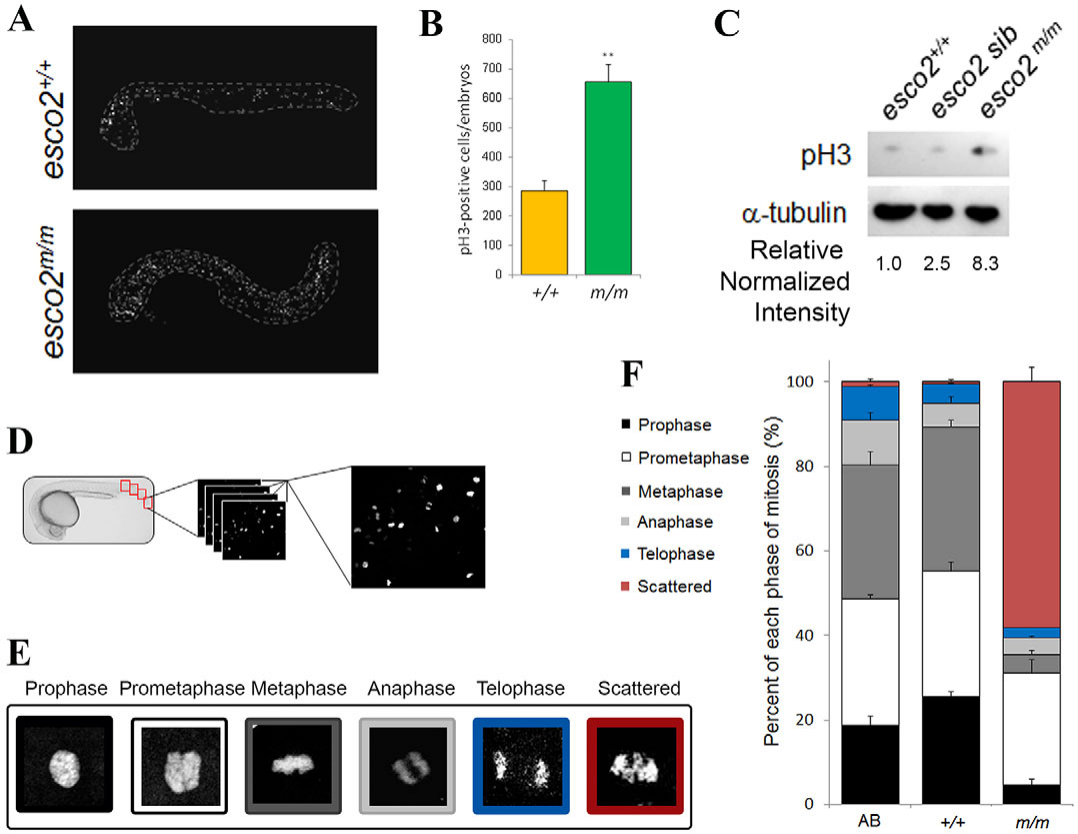
***esco2* deficiency results in an elevated mitotic index and scattered chromosomes.** (A) Maximum intensity projections of whole-embryo confocal *z*-stack images of pH3-stained fixed WT and mutant embryos. The embryo proper is outlined by the white dotted line. Yolk has been removed for imaging. (B) Quantification of the number of pH3-positive cells per embryo in A (*n*=3/genotype, mean±s.d., **P<0.01). (C) Western blot analysis for pH3 protein levels from *esco2^+/+^* (AB), *esco2* sibling (*^+/+^* and *^m/+^*) and *esco2* mutant (*^m/m^*) embryo protein lysates. Relative intensity calculated using ImageJ; each sample was normalized to α-tubulin intensity, and then relative expression was calculated against *esco2*^+/+^ (relative normalized intensity=1). (D) Diagram of mitotic profiling. Four independent fields of a pH3-labeled embryo are imaged, compiled using maximum intensity projection, and scored for each phase of mitosis based on chromosome morphology. (E) Colored panels depict the associated pH3 morphology to its phase in mitosis. (F) Graph depicting the percentage of cells in each phase of mitosis between AB (the WT parental strain) controls, *esco2^+/+^* and *esco2^m/m^* embryos (four fields/embryo, *n*≥70 morphologies per embryo, three embryos/genotype, mean±s.d.); the *P*-value of scattered between *esco2*^+/+^ and *esco2*^*m/m*^ is 0.0062.

SCC is required for proper mitotic progression. Toward determining the cellular consequences that lead to this embryonic lethality, we performed western blot as well as immunohistochemistry on WT and mutant embryos with an anti-phosphorylated histone-H3 (pH3; a marker of cells in M-phase of the cell cycle) antibody. A significant increase in the number of pH3-positive cells (2.3-fold; Fig. 3A,B) and total pH3 protein (8.3-fold; Fig. 3C) was observed in *esco2^m/m^* compared to WT embryos, suggesting that mutant embryos undergo mitotic arrest.

To determine at which phase of mitosis pH3-positive cells were accumulating, we generated mitotic-phase profiles of AB, *esco2^+/+^* and *esco2^m/m^* embryos. These were immunolabeled with pH3 and observed under confocal imaging. Four independent fields were imaged through the entire embryo to capture all cells in mitosis in a given field (Fig. 3D). Based on pH3 morphology, the phase of mitosis was determined for each pH3-positive cell and quantified for each genotype (Fig. 3E,F). Although all genotypes displayed the five distinctive mitotic phases (prophase, prometaphase, metaphase, anaphase and telophase), the *esco2^m/m^* embryos also displayed a unique scattered chromosome morphology (Fig. 3E,F). Although AB and *esco2^+/+^* profiles were comparable, the *esco2^m/m^* embryos had a significant increase in the number of scattered chromosome-containing cells (Fig. 3F). Within mutants, 60% of pH3-positive cells had the scattered morphology, which accounts for the increase in the total number of pH3-positive cells (Fig. 3B). These data suggest that the scattered phenotype results in a mitotic arrest leading to the early lethality in the *esco2^m/m^*. Furthermore, we did not observe a difference in the presence of scattered phenotype or the mitotic profile in a p53 mutant background (supplementary material Fig. S4), suggesting that, although p53 responds to this defect and loss of p53 temporarily abrogates the apoptotic response, it does not influence or rescue the actual mitotic defects in the *esco2* mutant embryos.

### Dynamic, in-embryo analysis of chromosome segregation reveals detailed consequences of Esco2 loss

**Fig. 4.**
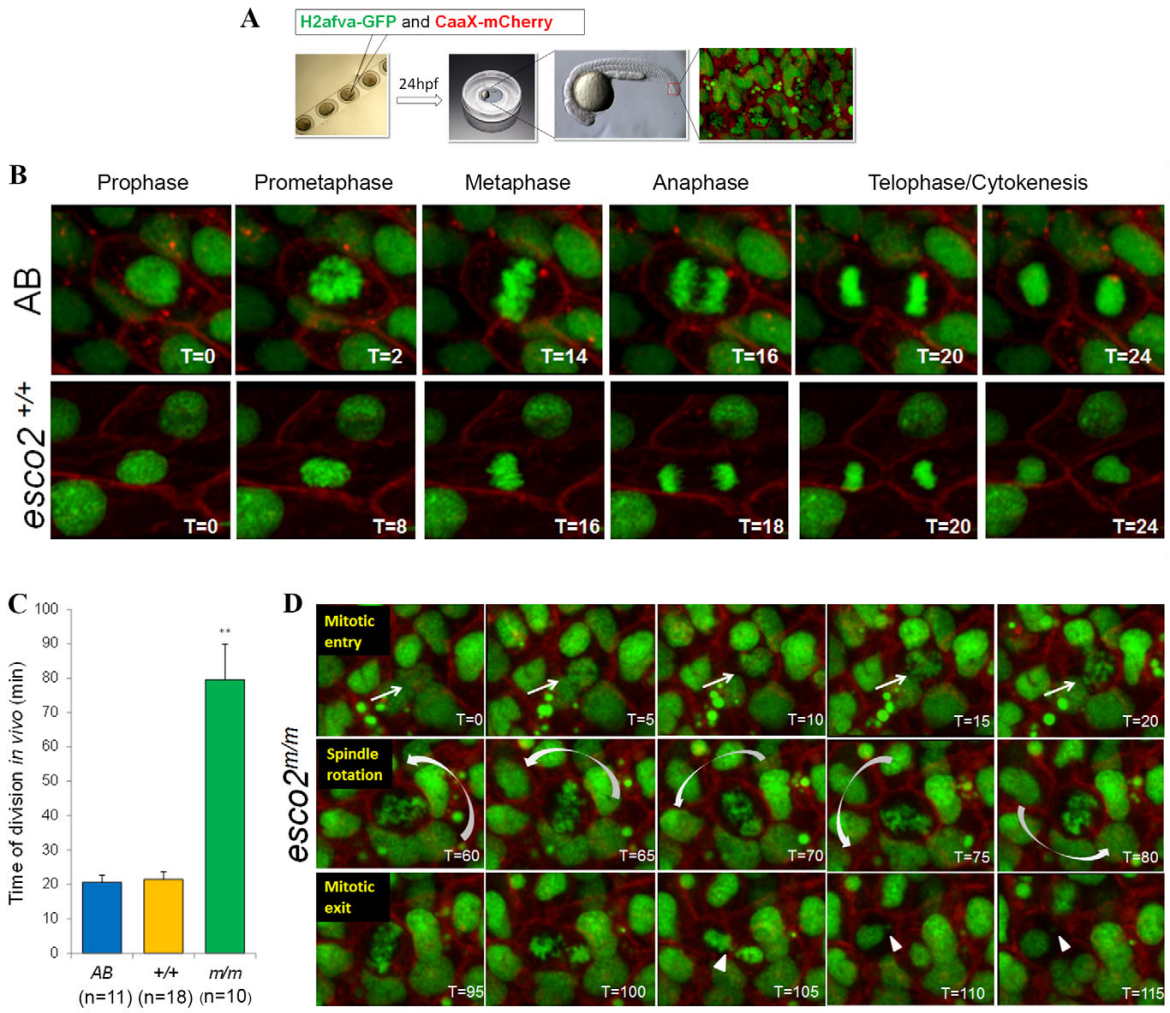
***In vivo* analysis of *esco2* mutants reveals chromosome scattering and a prolonged division time.** (A) Schematic of in-embryo confocal imaging. Embryos are injected at the one-cell stage, embedded in low-melt agarose at 24 hpf in a coverslip-bottom dish, and visualized with confocal imaging focusing on the thinner tail region. (B) Stills extracted from time-lapse imaging videos of wild-type embryos emphasizing phases of mitosis beginning at prophase and ending at the formation of two daughter cells. Time stamps are in minutes. (C) Division time of AB, *esco2^+/+^* and *esco2^m/m^* calculated from nuclear envelope breakdown (NEB) to division into two daughter cells in minutes (mean±s.d., **P<0.01 derived from comparing m/m to either AB or +/+). (D) Time-lapse imaging stills extracted from videos depict *esco2* mutant's mitotic entry, spindle rotation and scattering, and mitotic exit resulting in micronuclei formation (arrowhead). Arrows point towards the cell of interest. Curved arrows orient to the direction of spinning. Time stamps are in minutes.

The static analysis of mitosis thus far indicates that many cells in the *esco2^m/m^* embryos undergo a mitotic arrest with a scattered chromosome phenotype and suggests that this extended mitotic arrest leads to cell death during mitosis. To obtain a more dynamic analysis under physiological conditions, we have developed a novel technique to monitor chromosome segregation at the single-cell level within a live zebrafish embryo. One-cell-staged embryos are injected with mRNA encoding H2afva-GFP (labels chromatin) and CaaX-mCherry (labels plasma membrane). At 24 hpf, embryos are placed in a coverslip-bottom dish and imaged using time-lapse confocal microscopy (Fig. 4A).

With this approach, we demonstrate that AB and *esco2*^+/+^ embryos undergo normal progression through mitosis (Fig. 4B) with an average division time – from nuclear envelope breakdown (NEB) to nuclear envelope reformation (NER) in the two daughter cells – of 21 min (Fig. 4C; supplementary material Movie 3). However, cells from *esco2^m/m^* embryos undergo a rapid progression to the scattered chromosome phenotype following NEB (Fig. 4D; supplementary material Movie 4) and never form a proper metaphase plate. Upon chromosome scattering, the entire chromatin material proceeds to rotate within the cell for a prolonged period of time (Fig. 4D). To our surprise, the cells with scattered chromosomes do eventually divide with an average division time of 80 min (Fig. 4C). The longest complete mitosis from NEB to NER observed for a cell with scattered morphology was 2 h; however, a number of scattered cells persisted beyond the 4-h time-lapse recordings. This analysis suggests that loss of Esco2 results in chromosome scattering following NEB, which induces a prolonged mitotic delay most likely due to failure to satisfy the spindle assembly checkpoint.

From the *in vivo* imaging of embryos, we did not observe the formation of apoptotic bodies during mitosis; however, we did observe apoptosis occurring within interphase cells (supplementary material Movie 5). This suggests that the apoptotic event occurs after mitotic exit, potentially in G1, in which p53 has been strongly associated with an apoptotic response (Yonish-Rouach et al., 1993).

### Loss of Esco2 results in genomic instability

**Fig. 5.**
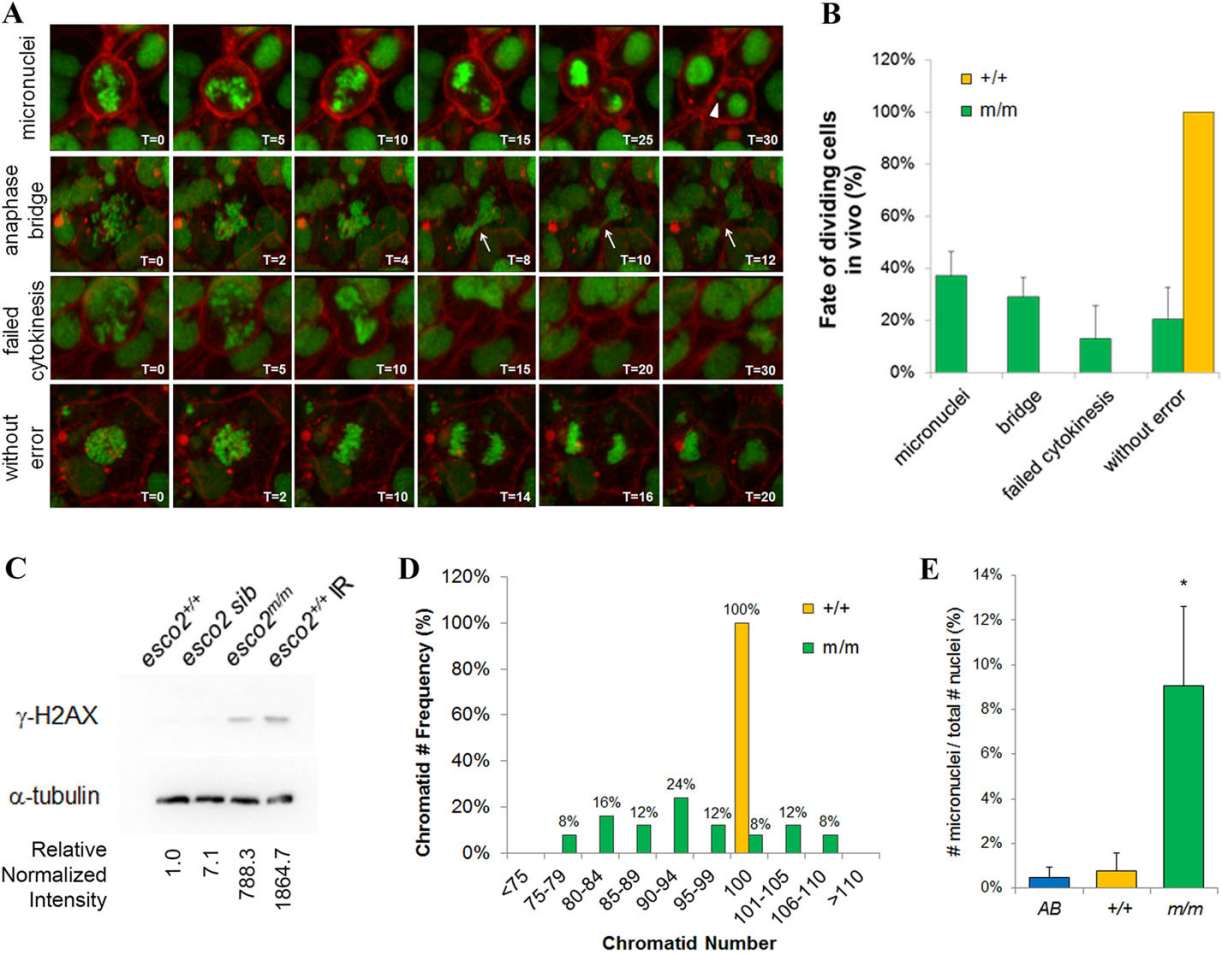
**Depletion of *esco2* results in genomic instability.** (A) Stills taken from time-lapse imaging videos demonstrating the variety of genomic instability observed in 24-hpf *esco2* mutant embryos. Micronuclei, anaphase bridges and failed cytokinesis were observed, as well as ‘without error’ divisions. Arrowhead points towards micronuclei. Arrow points to an anaphase bridge. Time stamps are in minutes. (B) Average frequency of above division fates in wild-type (11 divisions taken from three embryos) and *esco2* mutant (43 divisions from four embryos) embryos based on time-lapse imaging. Error bars show mean±s.d. between embryos. All wild-type cells underwent a normal division; therefore, there is no s.d. or error bar to report. (C) Western blot of γ-H2AX in protein lysates from 24-hpf *esco2^+/+^* (AB), *esco2* sib (^+/+^ and ^*+/m*^) and *esco2^m/m^* embryos. Irradiated (IR) embryos (100 Gy at 24 hpf and collected at 30 hpf) serve as a positive control. Relative intensity was calculated using ImageJ; each sample was normalized to α-tubulin intensity, and then relative expression was calculated against *esco2*^+/+^ (relative normalized intensity=1). (D) Quantification of the number of chromatids within metaphase spreads (*n*≥20 spreads per genotype) from pooled (10-12 embryos) *esco2^+/+^* (AB controls) and *esco2^m/m^* embryos. (E) Frequency of micronuclei observed in interphase cells of the tail region of embryos injected with *H2afva-eGFP; CAAX-mCherry* mRNA. Percentage is based on total number of micronuclei observed over the number of nuclei observed in interphase cells (*n*=3 embryos/genotype, >75 cells per field, mean±s.d., *P<0.05).

We observed, through in-embryo, time-lapse imaging, that, upon division, multiple *in vivo* segregation defects occur. Whereas, amongst WT embryos (three embryos, 11 divisions monitored), no erroneous divisions were observed, among 43 mutant divisions monitored from four different embryos, on average: 37% (±9% s.d.) of the divisions per embryo had lagging chromosomes [66% (*n*=10/15) involved one sister chromatid; 13% (*n*=2/15) involved two sister chromatids; 7% (*n*=1/15) involved three sister chromatids; 13% (*n*=2/15) involved 4+ sister chromatids] that developed into micronuclei [MN; Fig. 5A,B; supplementary material Movie 6; 85% (*n*=11/13) of cell divisions result in forming just one MN and 15% (*n*=2/13) of cell divisions result in two MN being formed]; 29% (±7% s.d.) of divisions per embryo had chromosomes decondense prior to cytokinesis, resulting in anaphase bridges during cytokinesis (Fig. 5A,B; supplementary material Movie 7); and 13% (±13% s.d.) of divisions per embryo had the chromosomes decondense without cytokinesis (Fig. 5A,B; supplementary material Movie 8) reminiscent of endoreduplication. We observed a significant increase in phosphorylated-H2AX (γ-H2AX) in mutants compared to AB or WT sibling controls (Fig. 5C), suggesting that chromosome-segregation abnormalities induce a DNA damage response. Interestingly, we observed that eight of the 43 (21%) divisions in *esco2^m/m^* embryos underwent what appears to be normal ‘without error’ mitotic divisions (Fig. 5A,B; supplementary material Movie 9).

To further address the consequence of these imprecise divisions, we analyzed our metaphase spreads from AB control and homozygous mutant embryos for the number of chromatids present. Zebrafish have 25 chromosomes and therefore 100 sister chromatids per metaphase cell. Most striking was that the majority (92%) of spreads from *esco2^m/m^* embryos did not have 100 chromatids (Fig. 5D). In fact, there was a bias toward loss of chromatids (73% loss versus 19% gained), suggesting that either the cells with micronuclei or the chromosome(s) contained in the micronuclei are eliminated. The reduction in the number of micronuclei in interphase cells (Fig. 5E), and our observation of a micronuclei-containing cell undergoing apoptosis (supplementary material Movie 5), further support the elimination of cells containing micronuclei. The chromosome numbers in *esco2^m/m^* ranged from loss of 25 chromatids to gain of ten chromatids (Fig. 5D). Because the majority of mis-segregation results in one mis-segregated chromosome per division, this suggests that the large range of chromatid numbers was the consequence of multiple defective divisions. These data suggest that it is not the aneuploidy (the change in chromosome number), but the micronuclei formation that is deleterious to a cell.

### Esco2 is required for cohesion establishment; however, compensatory cohesion mechanisms within some cells restore timely divisions and proper chromosome segregation in *esco2* mutant embryos

**Fig. 6.**
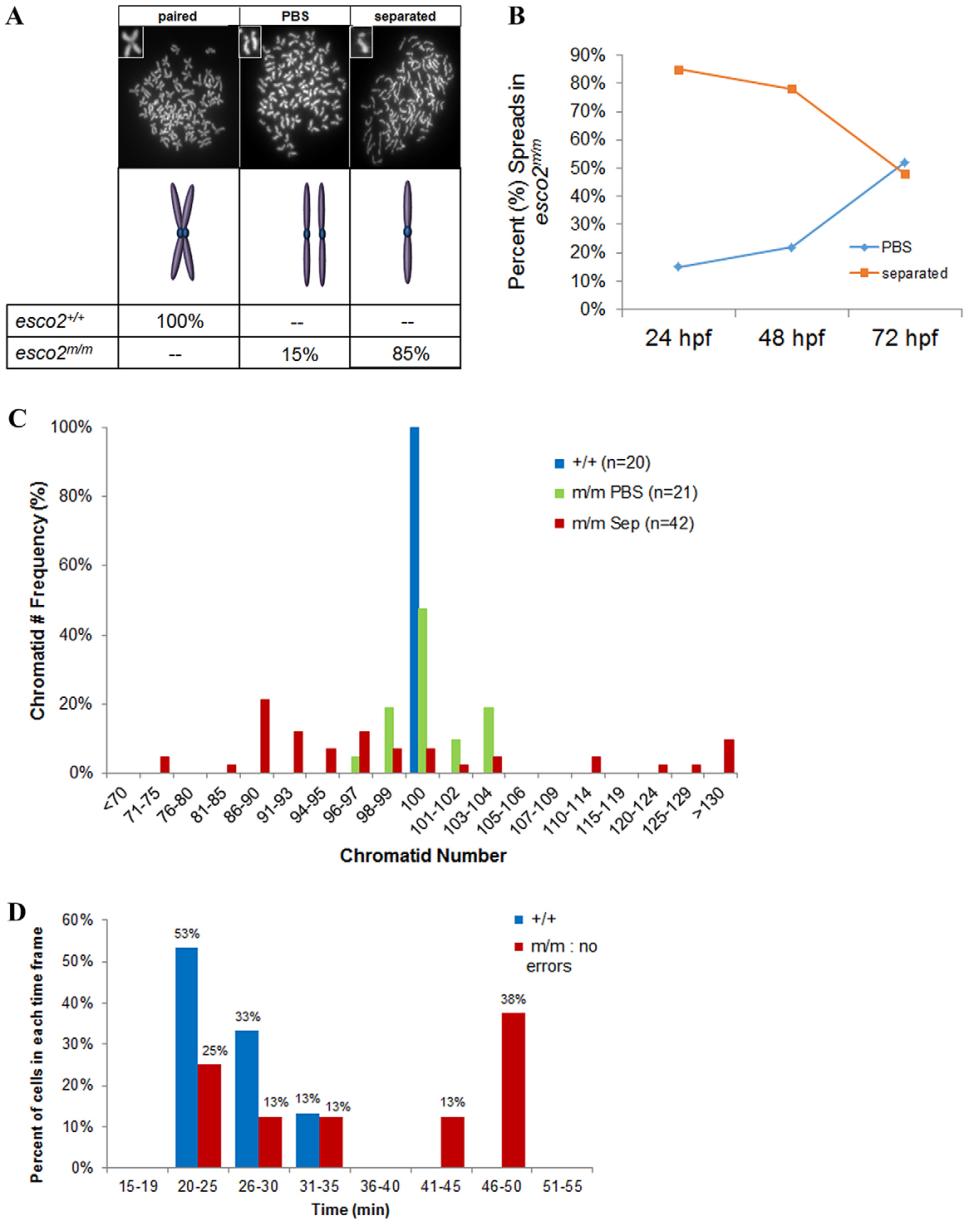
**Most cells of the *esco2*^*m/m*^ embryo display complete cohesion loss, although some cells display mild cohesion defects, mild aneuploidy and almost normal mitotic transition.** (A-C) Metaphase chromosome spreads from pooled (10-12 embryos) *esco2^+/+^* (AB) and *esco2^m/m^* embryos display three key categories: ‘paired’, ‘paired but separated’ (PBS) and ‘separated’. (A) Percentage distribution of spread categories (*n*≥20 spreads/genotype) from pooled *esco2^+/+^* and *esco2^m/m^* 24-hpf embryos. Insets in chromosome spreads are higher-magnified versions of the observed categories. If mixed categories were observed in the same spreads, they were counted toward the category in which the most prevalent phenotype was observed. (B) Frequency of PBS and separated spread categories from pooled *esco2^m/m^* at 24, 48 and 72 hpf (*n*≥20 spreads/time-point). (C) Frequency of chromatid number within a spread categorized to be either the ‘paired but separated’ (PBS) or ‘separated’ phenotype from 24-hpf pooled *esco2^m/m^* mutants. Chart also contains frequency of chromatid number from *esco2^+/+^* as a control. (D) Division time from NEB to NER of cells from *esco2^+/+^* embryos, or cells divisions deemed ‘without error’ from *esco2^m/m^* embryo time-lapse videos.

Both *esco1* and *esco2* (two homologs of yeast *eco1*) are responsible for establishing cohesion; thus, we wanted to determine whether loss of *esco2* alone would have an effect on cohesion between sister chromatids. Therefore, we generated metaphase spreads and observed three categories of metaphase spreads in WT and mutant embryos: (1) ‘paired’, SCC within the arms and the centromere; (2) ‘paired but separated’ (PBS) phenotype, in which the centromeres are separated but the sister chromatids still neighbor each other; and (3) ‘separated’, where sister chromatids are not cohered in the arms or centromere and appear as single chromatids (Fig. 6A). Although 100% of *esco2^+/+^* controls had the classic paired cohesion morphology, 85% of the *esco2^m/m^* spreads yielded a separated morphology, suggesting complete loss of cohesion in the absence of Esco2 and that the scattered phenotype is largely due to lack of cohesion between chromatids.

Interestingly, although the majority of *esco2* mutant spreads had complete chromatid separation, 15% of spreads yielded a PBS morphology. These data propose that there is only reduced cohesion within these cells and that alternative cohesion mechanisms exist to compensate for the loss of Esco2 in these cells. Alternatively, although the analysis of maternal *esco2* mRNA suggests that maternal transcripts are absent by 18 hpf (supplementary material Fig. S2) and published data indicate that the Esco2 protein is degraded during metaphase (therefore degrading maternal protein in early divisions) (Hou and Zou, 2005), there is the potential that, at 24 hpf, these 15% of cells with the PBS phenotype have a remnant of maternal *esco2*. Therefore, to determine whether this phenotype persists beyond normal maternal contributions, we have analyzed the amount of PBS spreads at 24, 48 and 72 hpf. Although the number of separated spreads decreases over time, presumably due to cell death or cellular arrest, the number of PBS cells increased (Fig. 6B).

To determine whether the PBS cells maintain a different ploidy, we analyzed chromatid number from separated and PBS spreads from *esco2* mutant embryos. The majority of spreads (92%) with ‘separated’ chromatids had improper ploidy (Fig. 6C). Remarkably, the sister chromatids that are partially separated in PBS spreads still achieve proper ploidy in 47% of PBS mitotic spreads, whereas 53% show only mild aneuploidy (Fig. 6C; ranging from ±4 chromatids). This would suggest that the spreads with proper ploidy in Fig. 5D are mostly the PBS cells. The high percentage of normal ploidy spreads (47%) suggests that multiple precise divisions must have occurred in these PBS cells. Therefore, mild separation does not seem to impinge greatly on microtubule attachment and segregation of sister chromatids at the metaphase-anaphase transition. These observations suggest that there are two pools of mutant cells: (1) cells with complete cohesion loss, separated chromatids, and high aneuploidy biasing toward loss of chromatids; and (2) weakened cohesion, PBS chromatids, and none to mild aneuploidy with equivalent gains and losses.

The presence of proper ploidy subsequently led us to hypothesize that the ‘without errors’ divisions that are present in ∼20% of *esco2* mutant cells (Fig. 5B) represents those cells that displayed the PBS phenotype in chromosome spreads. To determine whether there were differences in the division timing of these two populations of cells, we measured the timing from NEB to NER in 56 mitotic divisions in *esco2* mutant embryos using our in-embryo, time-lapse technique (Fig. 2A), with a 2-min interval between stacks over 2 h. The majority (73%) of divisions either: (1) underwent NEB but not NER, (2) were in the midst of dividing at the start of time-lapse, or (3) were in mitosis for the entire 2-h time-lapse. Because these were not complete divisions, accurate division times could not be determined and therefore were not included in this analysis. Complete divisions were segregated based on two categories: (1) those that display ‘genomic instability’ or (2) those that display division ‘without error’. Consistent with our previous observation on cell fates (20% in Fig. 5A,B), 14% (8 of 56) of the total amount of mitotic cells were ‘without error’. Importantly, 50% (4 of 8) of ‘without error’ cells underwent a comparable division time to WT cells (*t*=26.5 or 25.1 min, respectively), whereas the other 50% of ‘without error’ had an average division time of 47 min, twice the division time of *esco2* WT cells (Fig. 6D). We suggest that these divisions, although they undergo no evidence of genomic instability through live imaging, are delayed in satisfying the SAC. Although biased because it does not include the scattered genomic-instability-prone divisions that last the entire video time (15 of 56 divisions), the average division time for ‘genomic instability’ divisions was 92.8 min, much longer than the ‘without error’ divisions. Overall, these data suggest that, in an *esco2* mutant, a weakened cohesion phenotype exists in a subset of cells and that these cells divide with normal mitotic progression and ploidy.

## DISCUSSION

**Fig. 7.**
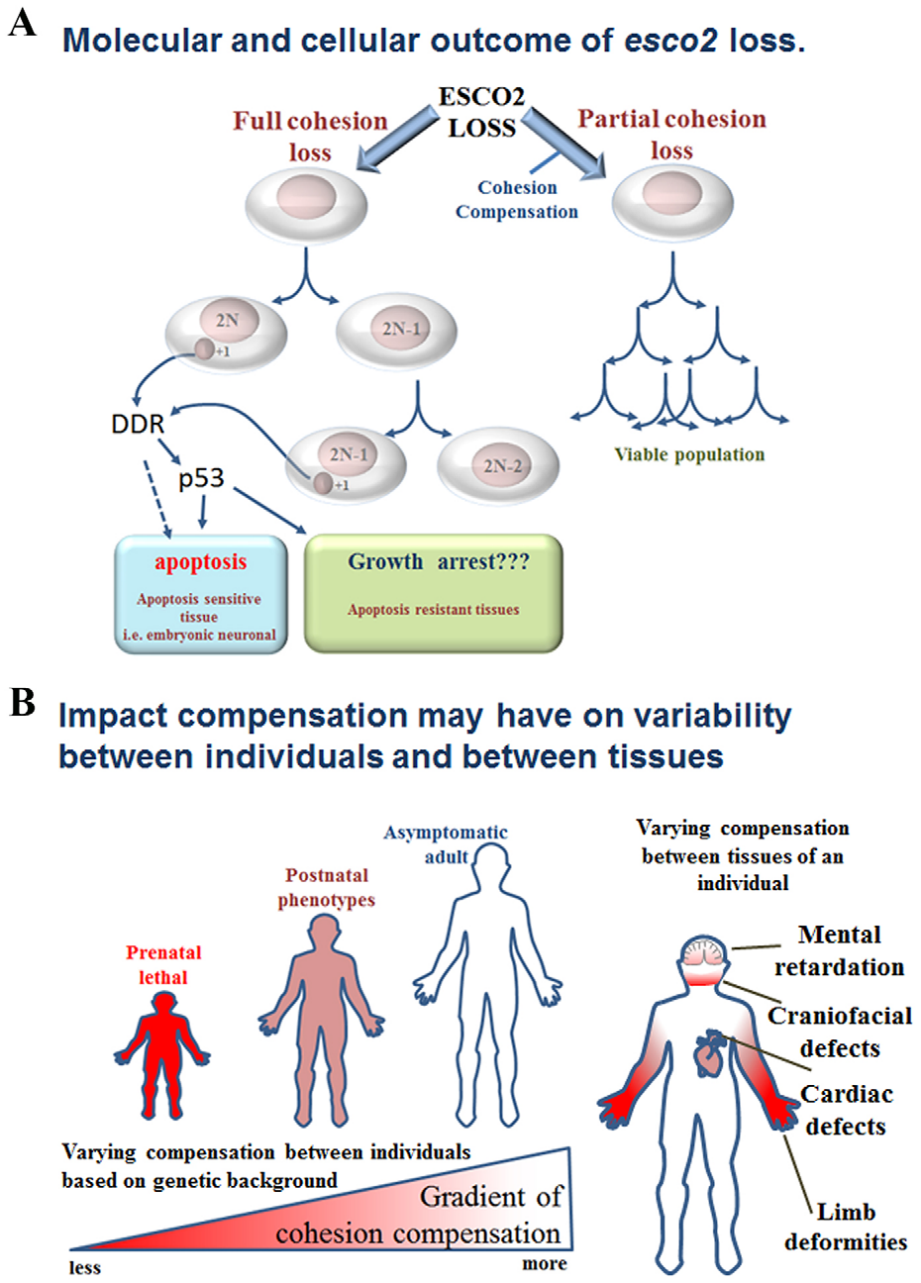
**Model depicting cellular outcomes in *esco2* mutant zebrafish embryos and hypothesized impact that compensation might have on RBS phenotypes.** (A) A model of the molecular and cellular event ongoing in the *esco2* mutant zebrafish. The majority of cells undergo a prolonged mitotic delay ultimately resulting in a single chromatid mis-segregation yielding a micronuclei. We observed: (i) through live imaging an interphase cell with a micronuclei undergo apoptosis, (ii) increased levels of γ-H2ax, indicating a DNA damage response (DDR), and (iii) p53-dependent apoptosis within the neural tube. Together, these data suggest that the micronuclei induce a DDR response to activate the p53 apoptotic response. There is subsequent apoptosis even in the absence of p53, suggesting that an alternative apoptotic signal exists. Other cells within the *esco2* mutant embryos appear to undergo a normal mitotic progression, and have only mild cohesion loss, suggesting that compensation mechanisms exist. (B) Depicts a model in which variation in compensation between individuals and between tissues in an individual might explain the phenotypic differences between individuals with RBS.

At the molecular level, our data indicates that, in the majority of cells, deficiency of *esco2* results in complete cohesion loss, genomic instability, aneuploidy and/or micronuclei formation that activates a DNA damage response that includes p53 (Fig. 7A). Our time-lapse imaging revealed that some cells containing micronuclei undergo apoptosis. Recent studies have demonstrated that micronuclei undergo late replication and induce a DNA damage response (γ-H2AX), which, in the absence of p53, can result in a chromothripsis phenomenon (Crasta et al., 2012). Our data is consistent with this, in that we observed micronuclei and γ-H2AX staining; however, in our mutants we also observed an apoptotic response that most likely reflects the presence of a functional p53. This suggests that it is the micronuclei, not aneuploidy, that is inducing a p53 response. It should be noted that, although we have focused on a lagging chromosome-micronuclei model (Fig. 7A), we have not truly addressed the amount of aneuploidy derived from the prematurely segregated chromosomes. Interestingly, our data (Fig. 6B) indicates that compensated cells (PBS) accumulate over time, whereas the number of cells with the ‘separated’ phenotype is reduced. This suggests that the ‘separated’/scattered cells undergo apoptosis, leaving a selective advantage of the PBS/compensated cell. Importantly, although p53 activation is a response to genomic instability, it does not correct the genomic instability, which ultimately cannot be tolerated by the organism; hence, there is only partial rescue of RBS phenotypes. With this in mind, we feel that therapies aimed at suppressing the stress response will only delay the defects considering that the stress is still present. Alternatively, the fact that we observe compensation in some cells, in addition to the genetic background influences on the severity of RBS in humans, suggests that, if we can restore cohesion, we can remedy many of the RBS phenotypes. Therefore, understanding the contribution that the genes in the cohesion network have on SCC *in vivo* becomes important.

We observed that the genomic-instability-induced apoptosis occurs predominantly in the neural tube. These results are consistent with other published data on DNA-damage-induced genomic instability in mouse and zebrafish (Lang et al., 2004; Berghmans et al., 2005; Sidi et al., 2008; Parant et al., 2010; Toruno et al., 2014). These tissue-specific effects might explain the many neural-related RBS phenotypes such as microcephaly, craniofacial defects and mental retardation. This observation is not unfounded in that, during embryonic development, persistent cellular stress (often due to inherited gene mutations that lead to cellular stress, i.e. genomic instability) results in p53 activation and neurodevelopmental phenotypes. Many of the neural-crest-derived craniofacial phenotypes in the Treacher Collins syndrome mouse model are resolved in a p53 mutant background (Jones et al., 2008). In addition, centrosomal stress has been linked to aneuploidy resulting in significant brain degeneration and microcephaly in a Plk4 overexpression mouse model (Marthiens et al., 2013). Studies of mosaic variegated aneuploidy (MVA) patients harboring mutations in Bub1b and Cep57 also showed aneuploidy and microcephaly (Hanks et al., 2006; Snape et al., 2011). Together, this suggests that early neuronal tissue might be more predisposed to undergo apoptosis in response to genomic instability. In fact, the lack of apoptosis at 48 and 72 hpf (Fig. 2C) and the morphological collapse of the head (Fig. 1C,F,G) suggest that all of the apoptosis-susceptible neural tube cells have died, while some scattered cells still exist (Fig. 6B).

Prior to identification of *ESCO2* as the gene responsible for RBS, there were two disorders: as a generalization, RBS encompassed the stillborn and early-postnatal-lethal individuals, whereas SC phocomelia syndrome was less severe and affected individuals often lived to childhood and even adulthood (Schüle et al., 2005). Although the phenotypes vary between individuals, premature chromatid separation is a hallmark among patients and was used as a diagnostic tool. These two syndromes were united under the title RBS upon identification that 100% of both disorders were due to ESCO2 mutations, with no phenotype-genotype correlation identified, suggesting that genetic background likely influences the phenotypes (Maserati et al., 1991; Schüle et al., 2005; Vega et al., 2010). Interestingly, in all animal models analyzed to date (yeast, fly, zebrafish and mouse), genetic loss of *ESCO2* results in early lethality (Skibbens et al., 1999; Toth et al., 1999; Williams et al., 2003; Whelan et al., 2012). This leaves the question, why are some human *ESCO2*-null individuals viable? In fact, one individual was identified to be *ESCO2*-null but displayed very mild phenotypes and therefore escaped diagnosis until adulthood (Goh et al., 2010). On the other side of the coin, there is the notion that humans null for *ESCO2* are not early embryonic lethal; however, there is an ascertainment bias toward identifiable patients (stillborn fetuses, children with birth defects and viable patients), but not embryonic lethal prior to the second term due to lack of detection. Part of the early lethality or lack of variable phenotypes amongst animal models can be attributed to the isogenic/near-isogeneic nature of the models. Alternatively, humans might have evolved compensation mechanisms, such as potentially higher levels of ESCO1. Whereas *Esco2*-null mice are early eight-cell (∼3 dpf) lethal, the zebrafish mutant proceeds through gastrulation and early embryogenesis (<18 hpf) owing to maternal stores of *esco2* mRNA. This is convenient in the sense that RBS-like phenotypes can be observed owing to the unabated transition through early embryogenesis. Interestingly, whereas our *esco2* mutant embryos displayed many of the severe RBS phenotypes, the published morpholino knockdown displayed milder RBS phenotypes, such as craniofacial defects (Mönnich et al., 2011). The morpholino is only a partial knockdown of *esco2*; therefore, this observation most likely reflects the *esco2* dose effect on phenotypes. Whether through compensation or *esco2* dose, this lends to our hypothesis that the amount of cohesion dysfunction will correlate with the severity of the RBS phenotypes (Fig. 7B). Although the above example focuses on *esco2*, involving the large number of SCC factors in the complex genetic network will expand our understanding of which factors influence the amount of cohesion dysfunction and how this network impacts the severity of disease.

The fact that we observed compensation in some cells opens up the possibility that there are other genetic factors (maybe *esco1*) that influence cohesion establishment, and these factors might have higher expression in a subset of cells/tissues (Fig. 7B). Toward this, we have recently generated an *esco1* mutant, which has cohesion defects as well as compensation in a subset of metaphase spreads (S.M.P., H.R.T. and J.M.P., unpublished data). This suggests that: (1) *esco1* is also important for cohesion establishment; and (2) *esco1* and *esco2* might have differential cell expressions, resulting in the variable cohesion loss. Beyond *esco1* and *esco2*, there are a number of cohesion establishment (e.g. ATAD5, CHTF18 and RBMX1), maintenance (e.g. SGOL1 and sororin) and anti-establishment factors (e.g. WAPAL, PDS5a, PDS5b and HDAC8) that might impinge on this compensation (Bermudez et al., 2003; Lengronne et al., 2006; Maradeo and Skibbens, 2009; Matsunaga et al., 2012). Clinically, this observation has the potential to explain why there are particular morphogenic phenotypes (i.e. limb deformities or craniofacial defects) amongst a normal body plan in individuals with RBS; some tissues might have differential compensation for ESCO2 loss.

In closing, we have identified a zebrafish genetic mutant in *esco2* that models RBS. The transparency of the zebrafish embryo has allowed us to monitor the *in vivo* chromosome segregation dynamics in real time and revealed the dynamic chromosome segregation defects in the *esco2* mutants. In addition, the future use of guide-directed EGFP-tethered endonuclease-dead Cas9 will allow for *in vivo* monitoring of the distances between sister chromatids (identified as two EGFP spots), much like that used in yeast cohesion separation studies (Straight et al., 1996; Michaelis et al., 1997; Chen et al., 2013). Applied to the *esco2* mutant embryos, this technique will help to spatially identify which cells have PBS and which are completely separated, in addition to determining the long-term consequence of these phenotypes. Furthermore, the ability of some cells to compensate for Esco2 loss suggests differential cohesion dysfunction between cells or tissues and might explain the specific RBS phenotypes. Importantly, understanding this compensation network has therapeutic application if cohesion can be restored in RBS patients and in individuals with other cohesion-driven diseases.

## MATERIALS AND METHODS

### Zebrafish lines

All zebrafish lines were maintained as described in Westerfield (1995) under standard laboratory conditions (Westerfield, 1995). AB WT zebrafish were used for morpholino injections and controls. The *esco2* retroviral insertion allele was obtained from Nancy Hopkins and Jacqueline A. Lees (Massachusetts Institute of Technology, Cambridge, MA) and maintained on the AB background.

### Genotyping

#### High resolution melt curve analysis (HRMA)

Individual embryos or tail clippings were placed in 100 µl ELB (10 mM Tris pH 8.3, 50 mM KCl, 0.3% Tween 20, 0.3% NP40, 1 mg/ml Proteinase K) in 96-well plates. Embryos/tail clips were incubated at 55°C for 4 h to overnight, depending on sample size, to generate genomic DNA. To inactivate Proteinase K, plates were incubated at 95°C for 10 min. For *esco2* hi2865 genotyping, PCR fragments were generated using primer V: 5′-TTTCACTGTTTCTGCAGGTTG-3′ and X: 5′-TAAGGTCTTCGAAGTCTTAACG-3′ to amplify WT products. Primers V: 5′-TTTCACTGTTTCTGCAGGTTG-3′ and W: 5′-GGGGGGGGGCCTACAGGTGGGGTCTTTC-3′ were used to amplify the viral insertion product. PCR reactions were performed using genomic DNA in black/white 96-well plates (Bio-Rad cat. no. HSP9665). PCR reaction protocol for retroviral insertion detection was 95°C for 20 s, then 40 cycles of 95°C for 10 s, 59°C for 20 s and 72°C for 8 s in Eppendorf Mastercycler Pro 96S. Following PCR, plates were analyzed for melting curves with Lightscanner (Idaho Technology) over a 65-95°C range. From this, WT, heterozygous and mutant melting temperatures were clearly distinguished. As previously published (Thomas et al., 2014), HRMA was used to identify CRISPR-derived *esco2* heterozygous mutant F1 zebrafish. HRM primers were 5′-GCTAGAATCTCCCCCAAAGC-3′ and 5′-AGGGGTTTCTGCTTGCTGTA-3′. Genomic PCR encompassing this region was sequenced from HRM-positive F1 fish, and then desired mutant (+13) fish were propagated into the F2 generation.

#### PCR gel genotyping

To confirm melt curve analysis, individual embryo PCR reactions corresponding to an *esco2*^+/+^ (WT), *esco2*^*hi2865/+*^ and *esco2*^*hi2865/hi2865*^ (mutant) melting curve were performed using primers 5′-ACTGCGGGAAAAGTGAGAGA-3′ and 5′-TGATTAATTTTTGCCCAGCAC-3′ for WT products. Primers 5′-ACTGCGGGAAAAGTGAGAGA-3′ and 5′-AAGGCACAGGGTCATTTCAG-3′ amplified the viral insertion product, which was run on a 2% agarose gel.

### Microinjection of antisense morpholino and *esco2* CRISPR

Injection of *p53* morpholino (MO) or *esco2* Cas9/guide RNA was performed on one-cell-stage zebrafish embryos at a concentration using 0.5 nl of 0.85 mM. Injected embryos were incubated at 28°C until the indicated stage and analyzed via brightfield microscopy. The sequence of *p53* MO used to target exon 2 splice donor site of the *p53* gene was 5′-CCCTTGCGAACTTACATCAAATTCT-3′. *Cas9* mRNA was transcribed from the linearized pT3TS-nCas9n plasmid (Addgene) using the mMessage mMachine T3 kit (Life Technologies). Each RNA was purified using the RNeasy Kit (Qiagen). The CRISPR guide RNA was synthesized using the MegaShortScript T7 Kit (Life Technologies) and purified using the MegaClear Kit (Life Technologies). RNA concentration was quantified using the Nanodrop spectrophotometer. For CRISPR/Cas9 injections, 150 ng/µl of *Cas9* mRNA and 30 ng/µl of RNA were used (Thomas et al., 2014).

### Quantitative RT-PCR

RNA was extracted from approximately 30 pooled *esco2*^+/+^ (AB), *esco2* sibling (sib; containing WT and heterozygous embryos) or *esco2^m/m^* (based on mutant phenotype at 24 hpf) embryos using Trizol reagent (Life Technologies), according to the manufacturer's suggested protocol. Each RNA sample was diluted to 10 ng/µl using RNase-free water, and cDNA was synthesized from each sample using the High Capacity cDNA Reverse Transcription Kit (Life Technologies). Primers and probes for both *esco2* (NM_001003872.1) (primers Y and Z in supplementary material Fig. S1) and *Gapdh* (NM_001115114.1) were obtained from Life Technologies, and RT-PCR analysis was performed for each cDNA sample using an ABI Prism 7900HT Fast Real-Time PCR System. Gene expression was then calculated using the comparative C_T_ method.

### Microscopy and image analysis

#### Gross morphology, heart and fin imaging

Embryos were placed in 0.4% tricaine to anesthetize and then in methyl cellulose for proper positioning at indicated time points. DIC images were taken for the heart and fin, and brightfield images were taken of the gross morphology. Heart and gross morphology images were taken using a Nikon AZ100 using the 2× objective 0.5 NA 4× digital zoom (heart) and 2× digital zoom (gross morphology). Images were processed using NIS Elements software. Fin images were taken on a Zeiss Axio Observer fluorescent microscope using a 10×0.2 NA objective and processed with Zen 2011 Blue software.

#### Head size and growth area measurements

*esco2* heterozygotes were crossed to generate mutants and analyzed at 24 hpf, 36 hpf, 48 hpf and 72 hpf. Mutants were identified by phenotype and isolated while *esco2* sibs were correspondingly isolated. At each time point, an individual embryo was placed in methylcellulose in lateral position. Embryos were imaged using Nikon AZ100 in at 2× objective and 2× digital zoom. Site of measurement for the head size was determined by drawing parallel lines corresponding to head direction and eye placement. From these lines, an additional perpendicular line that bisects the two parallel lines and the center of the eye was drawn. It was this line that was measured using the measure analysis tool in ImageJ using arbitrary units. Embryos were once again placed in methylcellulose in lateral positioning. Growth area measurements were obtained in a similar manner. The full embryo was outlined and the area of each embryo was quantified. Measurements were obtained by using the same measure tool in ImageJ using arbitrary units.

#### Apoptosis assay

Embryos were dechorionated using pronase as stated above and incubated in 10 µl/ml acridine orange for 1 h in the dark. Embryos were washed 5× for 5 min with E3 embryo water. For Fig. 2B, DIC and fluorescence images were taken on a Zeiss AxioObserver.Z1 using a 20× objective NA 0.4. Images were processed using Zen Pro 2011 and ImageJ. For Fig. 2C, fluorescence was observed using Nikon AZ100 using GFP filter at 2× objective and 2× digital zoom.

#### Whole-embryo phospho-H3 stain

Embryos staged at 24 hpf were dechorionated using 30 µl pronase (30 mg/ml; Sigma p5147)/1 ml E3 blue embryo water (5 mM NaCl, 0.17 mM KCl, 0.33 mM CaCl_2_, 0.33 mM MgSO_4_, 10^−5^% methylene blue). Embryos were incubated for 10 min in pronase and washed 3× with E3 blue embryo water to remove chorions. Embryos were then fixed in microtubule fixative (1× PBS, 37% formaldehyde, 8% glutaraldehyde, 1 M MgCl_2_, 100 mM EGTA, and 10% Triton X) at room temperature. After a 2-h block (1× PBS, DMSO and 10% sheep serum), embryos were incubated overnight with anti-phospho-H3 (ser10) (Santa Cruz Biotechnology, sc-8656-R) at 1:200 dilution. Embryos were rinsed in block 3× for 20 min and then incubated in corresponding secondary antibody, goat anti-rabbit Alexa Fluor 647 (Invitrogen, a21245). Embryos were washed in 1× TBST and placed in slowfade (Invitrogen) until imaged. All embryos were deyolked using 27-gage needles prior to imaging.

#### Whole-embryo phospho-H3 imaging

Yolks were removed after fixation for imaging purposes. Whole-embryo *z*-stack (1.5 µm interval) confocal imaging was generated using a 5×0.12 NA objective, and on a Leica SP2 upright confocal microscope. Phosphor-H3 (ser10) embryos were quantified using ImageJ ICTN plugin.

#### Mitotic profiling

Four non-overlapping fields were imaged per embryo by taking a 1.5-µm *z*-stack through whole embryo using the Nikon A1R confocal microscope using 60×1.4 NA objective. Images were compressed and converted to black and white for ideal counting/detection conditions. Using morphology of pH3 staining, the phases of each cell were determined and quantified for each field (see Fig. 3E). This procedure was done for three embryos of each genotype. Percentages for each phase were quantified for each embryo, generating average percentage of each phase/genotype.

#### Time-lapse imaging

*CaaX-mCherry* and *H2afva-EGFP* mRNA was transcribed from a plasmid [pCS2-CaaX-mCherry and pCS2-H2afva-EGFP; gift from K. Kwan (University of Utah)] using mMessage mMachine SP6 kit (Life Technologies). *esco2* heterozygotes were crossed and embryos were microinjected into the yolk of a one-cell-staged embryo with 1 nl of 200 ng/µl *Caax-mCherry* and 200 ng/µl *H2afva-eGFP* mRNA. At 24 hpf, embryos were screened for fluorescence. Embryos were manually dechorionated using tweezers and anesthetized using 0.4% tricaine. In a glass-coverslip-bottomed dish, embryos were embedded in a 1% low-melt agarose. Dishes were placed on the Nikon A1 inverted confocal microscope, and *z*-stack images were taken at designated intervals. For AB and *esco2* WT videos, 40-µm *z*-stacks (with a 3-µm interval) were obtained every 2 min for a total scanning time of 2 h. Because *esco2* mutants have a dramatically longer division time, adjustments had to be made to account for photobleaching and to capture full *esco2* divisions from NEB to NER. *Z*-stacks were taken every 5 min for a total scanning time of 4 h. All videos were taken using 60×1.4 NA objectives. 3D viewing, still shots and videos were assembled and processed using NIS Elements 4.13.00.

#### Micronuclei/apoptotic bodies count

Embryos were injected with *H2afva-EGFP* and *CaaX-mCherry* mRNA and set up as if for a time-lapse video. To ensure consistency, for each field, a 40-µM *z*-stack was generated with 2-µm steps using 60×1.4 NA objective and 1.5 digital zoom on a Nikon A1 confocal microscope. Using 3D volume rendering in NIS Elements 4.13.00, an average nuclei, micronuclei and apoptotic body count was calculated per field to generate the percent observed in a population of cells. Filters for apoptotic bodies and micronuclei (versus nuclei) were set at H2afva-EGFP fluorescing body ≤3 µm. Differentiation between apoptotic bodies and micronuclei was determined based on size and localization within the cell; i.e. using the CaaX-mCherry (plasma membrane) fluorescence, it can be determined whether a micronuclei is within a cell and whether an apoptotic body is outside a cell. Frequency of micronuclei in interphase (Fig. 5E) was calculated by dividing the total number of micronuclei observed in the 3D render by the number of nuclei identified in the 3D render.

### SDS-PAGE and western blot analysis

Cell lysates for immunoblotting were prepared using 18-hpf, 24-hpf and 30-hpf embryos. Embryos were dechorionated using pronase procedure as stated above. Deyolking was performed by adding 200 µl deyolking buffer (55 mM NaCl, 1.8 mM KCl, 1.25 mM NaHCO_3_), pipetting up and down three times with a p200 pipette tip and then centrifuged for 2 min at 300 rpm. Supernatant was discarded and the above step was repeated once more. 60 µl protein prep (30 µl Invitrogen NuPAGE LDS sample buffer, 10 µl proteinase inhibitors, 1.5 µl β-mercaptoethanol, 18.5 µl water) was added to embryos and put on heat block at 95°C for 5 min. Lysates were microcentrifuged and put on heat block for an additional 5 min. Supernatant was transferred to a separate tube and stored at −20°C. Protein was loaded onto a 4-12% NuPAGE gel (Invitrogen) and transferred to a PVDF membrane. α-tubulin at 1:7000 (Abcam, ab7291-100) and GAPDH at 1:5000 (Cell Signaling, 2118S) were used as loading controls. Antibodies used were against pH3 at 1:5000 (Santa Cruz Biotechnology, sc-8656-R), p53 at 1:1000 (GeneTex, GTX128135) and γ-H2AX at 1:1000 (GeneTex, GTX127340). All blots were treated with Lumigen PS-3 detection agent. The pH3 and p53 blots were exposed to film, developed using the Konica SRX-101A system and imaged using the CareStream 212 Pro imaging system. The γ-H2AX blot was imaged using the Bio-Rad ChemiDoc MP imaging system. All digital images were scanned at 600 dpi and quantified using ImageJ.

### Chromosome spreads

Chromosomes spread protocol was adapted from the Lee group (Jeong et al., 2010). Approximately 20-30 embryos were dechorionated at 24 hpf. Embryos were incubated in 400 ng/ml nocodazole for 2 h in the dark at room temperature. Embryos were then transferred to 1.1% sodium citrate in a 6-cm dish. At this point, for genotyping purposes, tails were removed to be genotyped, whereas the remaining embryo heads were transferred to fresh sodium citrate solution and incubated on ice for 8 min. Next, we performed two washes with a cold 3:1 methanol:acetic acid solution for 20 min each followed by storage in −20°C until genotyping was performed. After fixative procedure, embryos were pooled (10-12 embryos/pool) per genotype and then minced using forceps in a 1:1 methanol:acetic acid solution. Using this mixture, 50 µl of pooled embryos were dropped onto a slide, and 3-5 drops of glacial acetic acid was added. The slide was slowly placed slide up and exposed to hot vapors (we used boiling water) for about 10 s; then the slide was allowed to dry on a hot metal surface (approx. 50°C). After the slide was completely dry, a few drops of Prolong Gold with DAPI were added and covered with a glass coverslip. Chromosomes were imaged using a 63×1.4 NA objective on a Zeiss Axio Observer fluorescent microscope and processed with Zen 2011 Blue software. Although most spreads were clearly delineated into the ‘paired’, PBS or ‘separated’ categories, if a spread had multiple phenotypes it was categorized by which was most prevalent in that spread. Chromatid number was counted manually from high-resolution images.

### Statistical analysis

Excel software was used in the generation of all graphs and statistical tests. Overall statistical significance was calculated using an unpaired *t*-test with error bars indicating s.d. as stated in legend (±). All *P*-values were determined significant at *P*<0.05. Unpaired *t*-test determined the significantly different values. Log-rank test determined significance in Kaplan–Meier survival curve analysis. Significance values are stated in the figure legends.

## Supplementary Material

Supplementary Material
