# Supplementary material for: Variations in dysfunction of sister chromatid cohesion in esco2 mutant zebrafish reflect the phenotypic diversity of Roberts syndrome
Source: Dis Model Mech. 2015 Aug 1;8(8):941–55. doi: 10.1242/dmm.019059 (PMC4527282; doi:10.1242/dmm.019059)
Supplement: Supplementary Material [file supp_8_8_941__index.html]

Supplementary Material 

# Variations in sister chromatid cohesion dysfunction in *esco2* mutant zebrafish reflects the phenotypic diversity of Roberts Syndrome

## DMM019059 Supplementary Material

- Supplementary Material
