# Supplementary material for: Variations in dysfunction of sister chromatid cohesion in esco2 mutant zebrafish reflect the phenotypic diversity of Roberts syndrome
Source: Dis Model Mech. 2015 Aug 1;8(8):941–55. doi: 10.1242/dmm.019059 (PMC4527282; doi:10.1242/dmm.019059)
Supplement: Supplementary Material [file supp_019059_DMM019059supp.pdf]

SUPPLEMENTAL VIDEOS AND FIGURES:

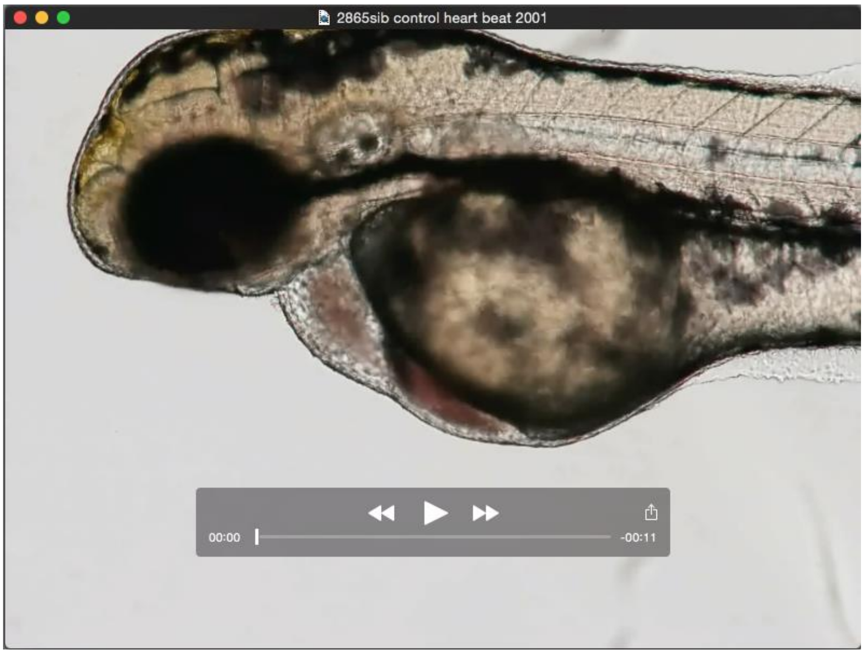

Video 1: *esco2*$^{+/+}$ heart beat is normal. Time-lapse imaging of heart beat in 2 dpf *esco2*$^{+/+}$ embryo. Imaging was performed on Nikon AZ100 using the 2x objective NA 0.5 and 4x digital zoom. Image was focused on heart and a video file was recorded for 10 seconds.

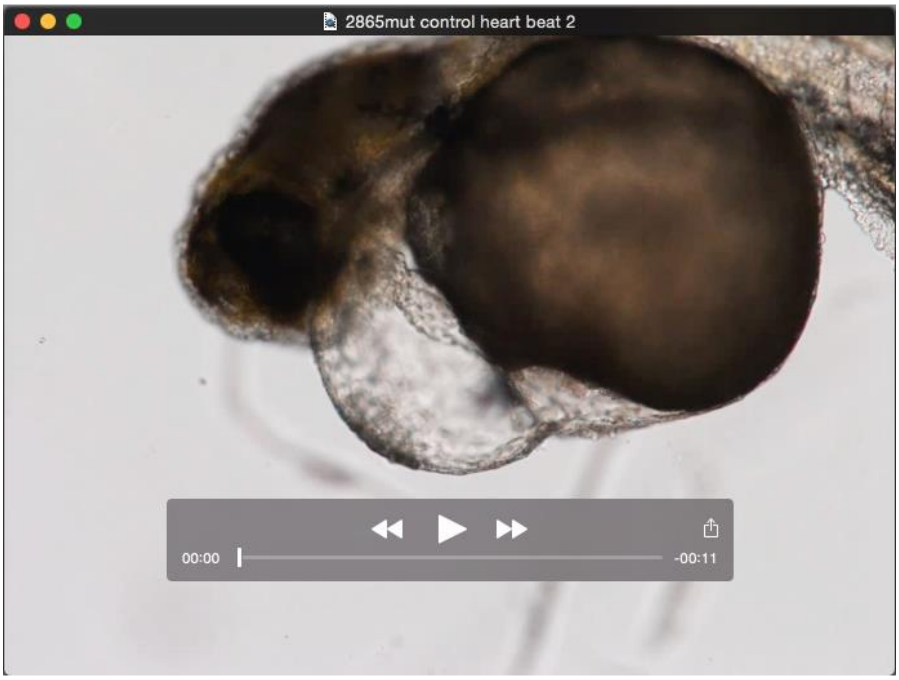

Video 2: *esco2$^{m/m}$* shows absence of heart looping but proper 1:1 A/V contractions. Time-lapse imaging of heart beat in 2 dpf *esco2$^{m/m}$* embryo. Imaging was performed on Nikon AZ100 using the 2x objective NA 0.5 and 4x digital zoom. Image was focused on heart and a video file was recorded for 10 seconds.

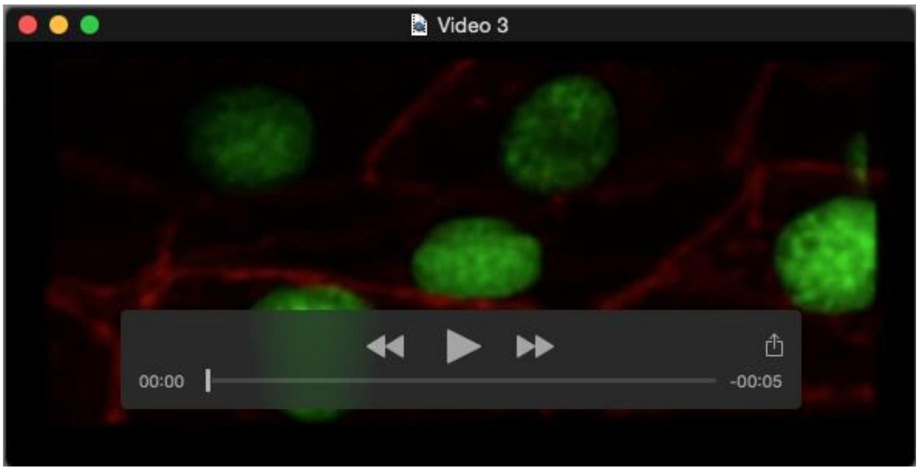

Video 3: *esco2*$^{+/+}$ division shows natural progression through mitosis. Time-lapse imaging of mitosis in an *esco2*$^{+/+}$ 24 hpf zebrafish embryo in which chromatin is labeled green and cell membrane red via H2afva-EGFP and CAAX-mCherry, respectively. NEB is noted by the irregular appearance of the nucleus and proceeds towards congression of the sister chromatids to form a metaphase plate. Accurate segregation occurs and results in formation of two new daughter cells. This particular division was completed in 24 minutes. Video was acquired on a Nikon A1 confocal microscope using a 63x objective. Z-stack of embryo tail was performed through approximately 40µm of tissue, obtaining a z-slice every 3µm. A z-stack was taken every 2 minutes for 2 hours. Z-stacks were compiled into a 3D projection where individual divisions were cropped out and put in sequence to show as time-lapse.

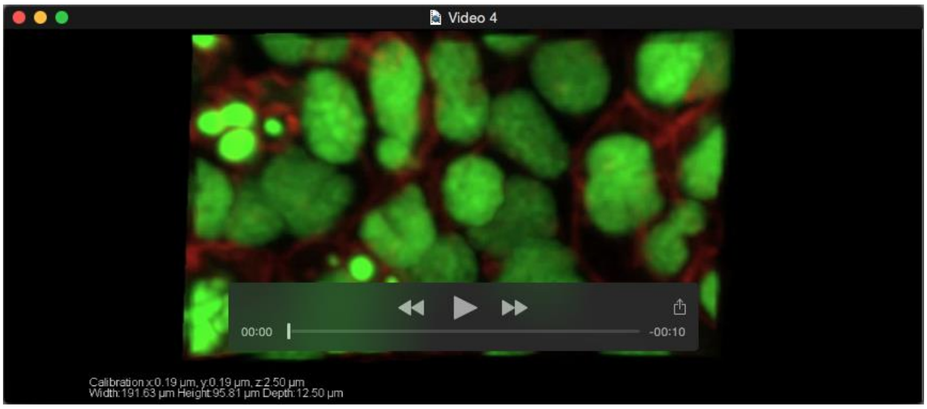

Video 4: *esco2$^{m/m}$* division displays chromosome scattering, spindle rotation and genomic instability. Time-lapse imaging of mitosis in an *esco2$^{m/m}$* 24 hpf zebrafish embryo in which chromatin is labeled green and cell membrane red via H2afva-EGFP and CAAX-mCherry, respectively. NEB is noted by the irregular appearance of the nucleus, however after NEB, chromosomes immediately scatter and begin to rotate on spindle axis. Within this video, the spinning persists for approximately 35 minutes before division which results in a lagging chromosome that forms micronuclei in the new daughter cell to the left. This division was completed in 115 minutes. Video was acquired on a Nikon A1 confocal microscope using a 63x objective. Z-stack of embryo tail was performed through approximately 40µm of tissue, obtaining a z-slice every 3µm. A z-stack was taken every 5 minutes for 4 hours. Z-stacks were compiled into a 3D projection where individual divisions were cropped out and put in sequence to show as time-lapse.

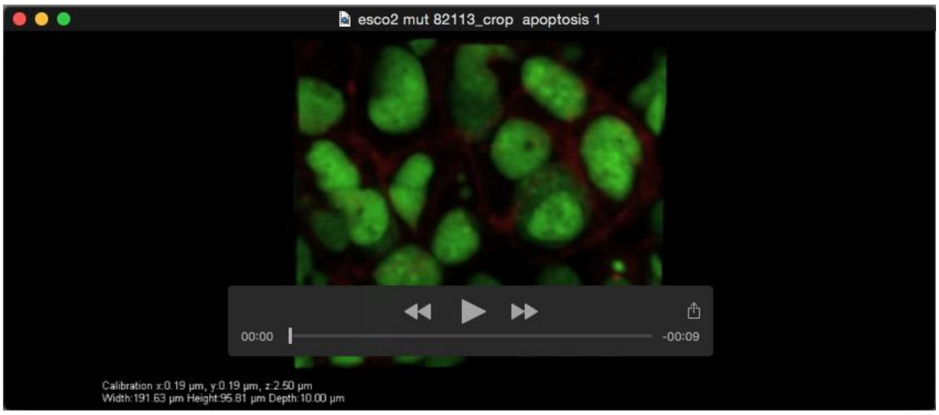

Video 5: ***esco2$^{m/m}$*** **division containing micronuclei ultimately leads to apoptosis.** Time-lapse imaging of mitosis in an e*sco2$^{m/m}$* 24 hpf zebrafish embryo in which chromatin is labeled green and cell membrane red via H2afva-EGFP and CAAX-mCherry, respectively. Video begins showing a cell in interphase with micronuclei. In this video, the cell portrays classic apoptotic morphologies such as the micronuclei and nucleus condense, cell size shrinks, membrane blebbing occurs, and eventually several apoptotic bodies form. Video was acquired on a Nikon A1 confocal microscope using a 63x objective. Z-stack of embryo tail was performed through approximately 40µm of tissue, obtaining a z-slice every 3µm. A z-stack was taken every 5 minutes for 4 hours. Z-stacks were compiled into a 3D projection where individual divisions were cropped out and put in sequence to show as time-lapse.

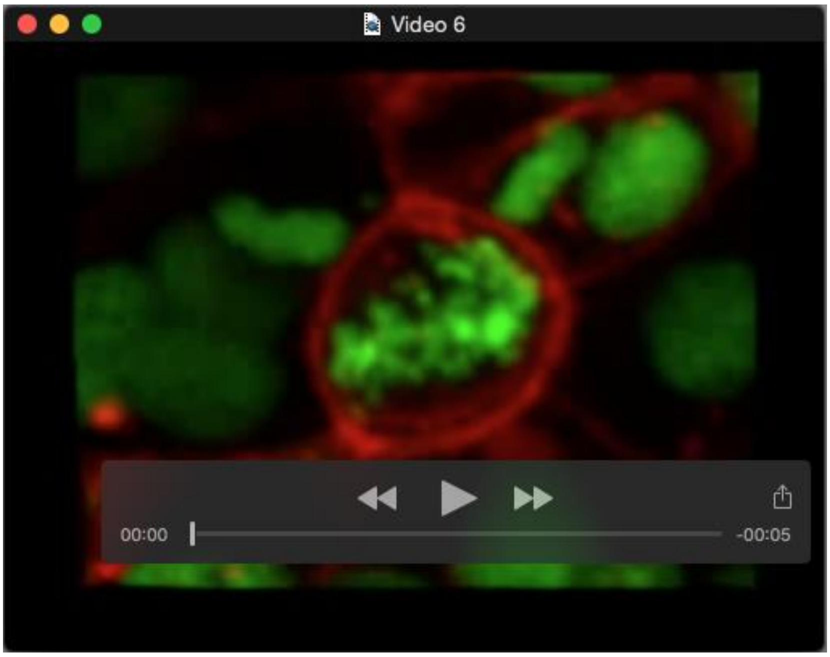

Video 6: *esco2*$^{m/m}$ division predominantly results in micronuclei formation. Time-lapse imaging of mitosis in an *esco2*$^{m/m}$ 24 hpf zebrafish embryo in which chromatin is labeled green and cell membrane red via H2afva-EGFP and CAAX-mCherry, respectively. Video begins with chromosomes in scattered morphology, which upon divides displaying a lagging chromosome that will result in micronuclei formation in the new daughter cell off to the left. Video was acquired on a Nikon A1 confocal microscope using a 63x objective. Z-stack of embryo tail was performed through approximately 40µm of tissue, obtaining a z-slice every 3µm. A z-stack was taken every 5 minutes for 4 hours. Z-stacks were compiled into a 3D projection where individual divisions were cropped out and put in sequence to show as time-lapse.

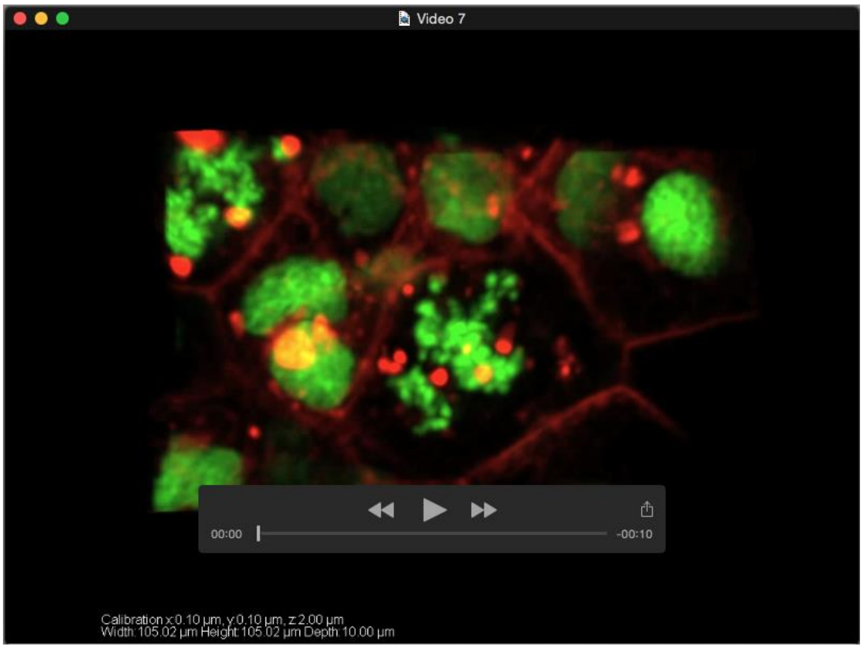

Video 7: *esco2$^{m/m}$* division results in anaphase bridge formation. Time-lapse imaging of mitosis in an *esco2$^{m/m}$* 24 hpf zebrafish embryo in which chromatin is labeled green and cell membrane red via H2afva-EGFP and CAAX-mCherry, respectively. Video begins with chromosomes in scattered morphology, but progresses to the point where the chromatin prematurely decondenses prior to accurate segregation of sister chromatids. However, cytokinesis still proceeds in which the cleavage furrow impinges on the decondensed chromosomes and, as abscission occurs, pulls the chromatin material to form an anaphase/nucleoplasmic bridge. Video was acquired on a Nikon A1 confocal microscope using a 63x objective. Z-stack of embryo tail was performed through approximately 40µm of tissue, obtaining a z-slice every 3µm. A z-stack was taken every 2 minutes for 2 hours. Z-stacks were compiled into a 3D projection where individual divisions were cropped out and put in sequence to show as time-lapse.

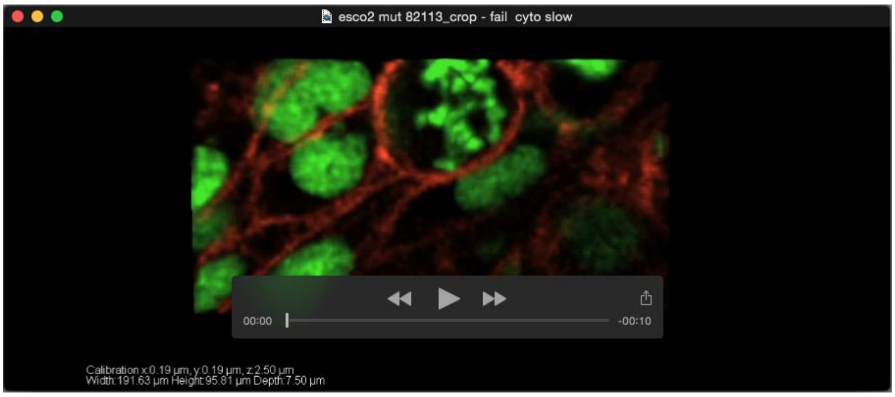

Video 8: *esco2$^{m/m}$* division results in failed cytokinesis. Time-lapse imaging of mitosis in an *esco2$^{m/m}$* 24 hpf zebrafish embryo in which chromatin is labeled green and cell membrane red via H2afva-EGFP and CAAX-mCherry, respectively. Video begins with chromosomes in scattered morphology, but progresses to the point where the chromatin decondenses without cytokinesis. Video was acquired on a Nikon A1 confocal microscope using a 63x objective. Z-stack of embryo tail was performed through approximately 40µm of tissue, obtaining a z-slice every 3µm. A z-stack was taken every 5 minutes for 4 hours. Z-stacks were compiled into a 3D projection where individual divisions were cropped out and put in sequence to show as time-lapse.

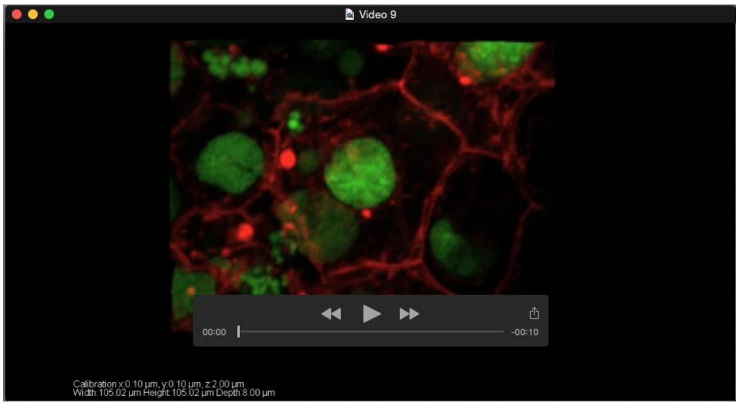

Video 9: *esco2$^{m/m}$* division results in a few normal mitotic divisions. Time-lapse imaging of mitosis in an *esco2$^{m/m}$* 24 hpf zebrafish embryo in which chromatin is labeled green and cell membrane red via H2afva-EGFP and CAAX-mCherry, respectively. Surprisingly, 20% of *esco2$^{m/m}$* divisions divide with natural progression and appear to have accurate segregation as in the *esco2$^{+/+}$*. Video was acquired on a Nikon A1 confocal microscope using a 63x objective. Z-stack of embryo tail was performed through approximately 40µm of tissue, obtaining a z-slice every 3µm. A z-stack was taken every 2 minutes for 2 hours. Z-stacks were compiled into a 3D projection where individual divisions were cropped out and put in sequence to show as time-lapse.

SUPPLEMENTAL FIGURES:

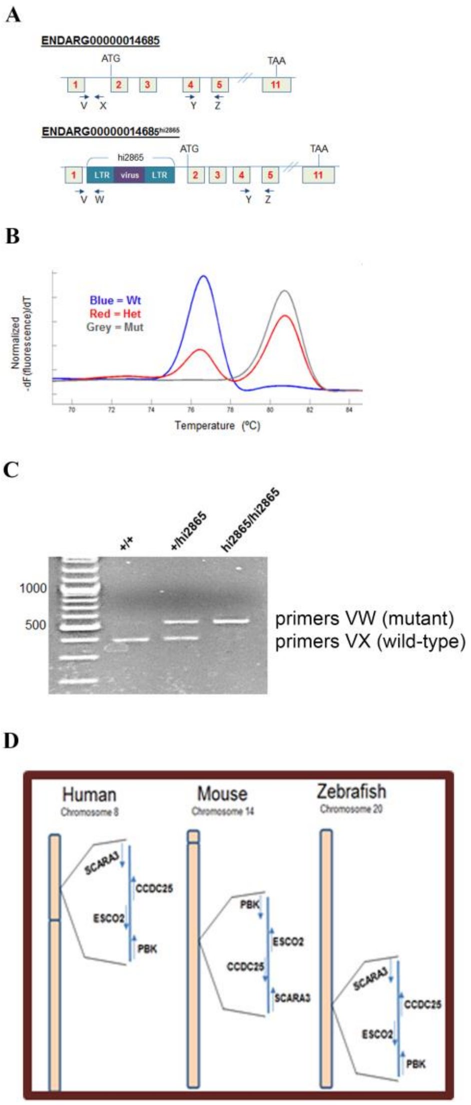

Supplemental Figure S1: Genomic analysis indicates that the hi2865 zebrafish embryonic lethal phenotype is due to insertion in the esco2 gene. (A) Illustration of ENDARG00000014685 transcript and the hi2865 retroviral insertion location in intron 1. ATG denotes the transcriptional start site and TAA denotes translation stop site. Primers V, W, and X were used for genotyping and Primers Y and Z used to quantitate mRNA levels. **(B)** High Resolution Melt curve analysis was used to genotype *esco2* alleles. **(C)** PCR gel electrophoresis initially used to genotype *esco2* alleles. **(D)** *esco2* gene synteny and organization between human, mouse, and zebrafish in highly conserved.

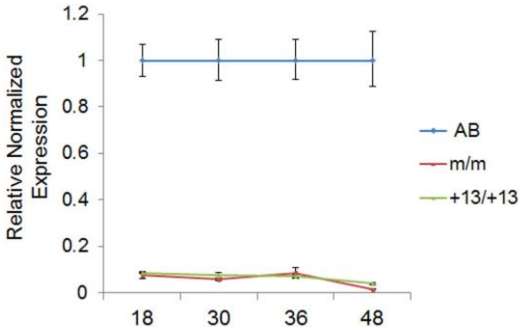

Supplemental Figure S2: *esco*2 mRNA is reduced >95% by 18 hpf in *esco2*$^{\mathbf{m/m}}$ embryos. qRT-PCR of *esco2*$^{+/+}$ (AB), *esco2*$^{m/m}$, and *esco2* $^{+13/+13}$ allele (see Supp. Fig. 3) at 18, 30, 36, and 48 hpf. Expression levels normalized to GAPDH and made relative to AB normalized expression. Note the *esco2* null allele must undergo nonsense mediated decay.

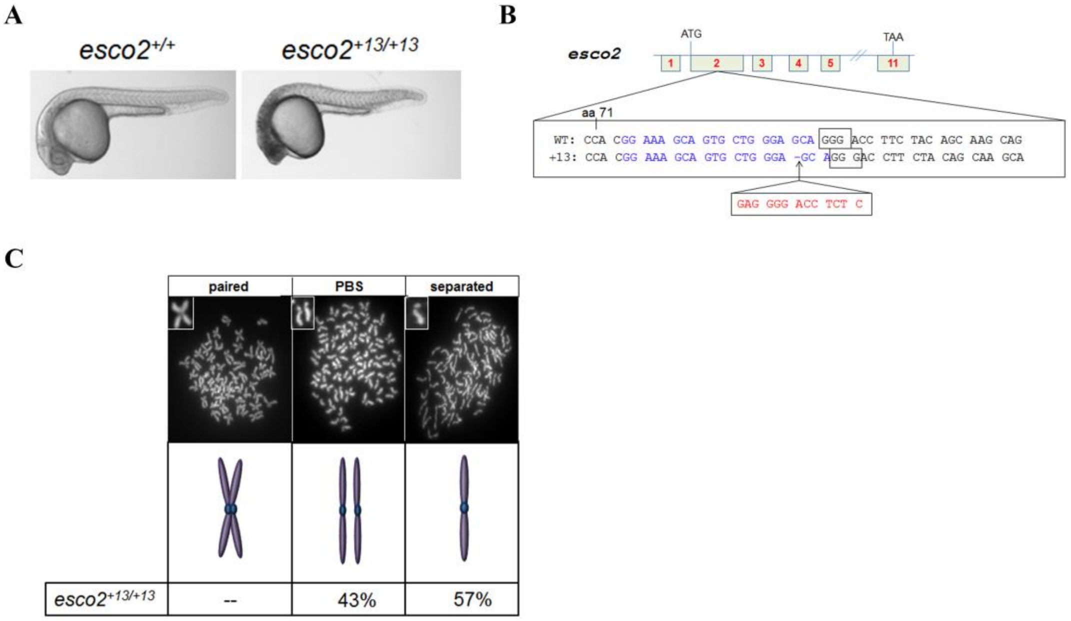

Supplemental Figure S3: CRISPR/Cas9 derived *esco2* null allele recapitulates *esco2* retroviral insertion mutant.

(A) Brightfield images of 24 hpf *esco2*$^{+13/+13}$ allele recapitulates embryonic lethality and head necrosis (n=41 with head necrosis and 137 WT morphology) of *esco2*$^{m/}$m. **(B)** Allele diagram of *esco2* +13 allele. A 13 bp insertion occurs in the second ATG containing exon of the 11 exon gene. ATG denotes the transcriptional start site and TAA denotes translation stop site. Blue text indicates CRISPR target site, the PAM is boxed, and red text indicates 13 bp insertion. Codons separated by spaces, note alternative codons in the +13 allele due to the frame shift. **(C)** Metaphase chromosome spreads from 24 hpf pooled (n=30) *esco2*$^{+13/+13}$embryos. Percentage distribution of each category: "paired", "paired but separated" (PBS), and "separated". Representative spreads and diagrams to describe category shown above percentages (n=30 spreads).

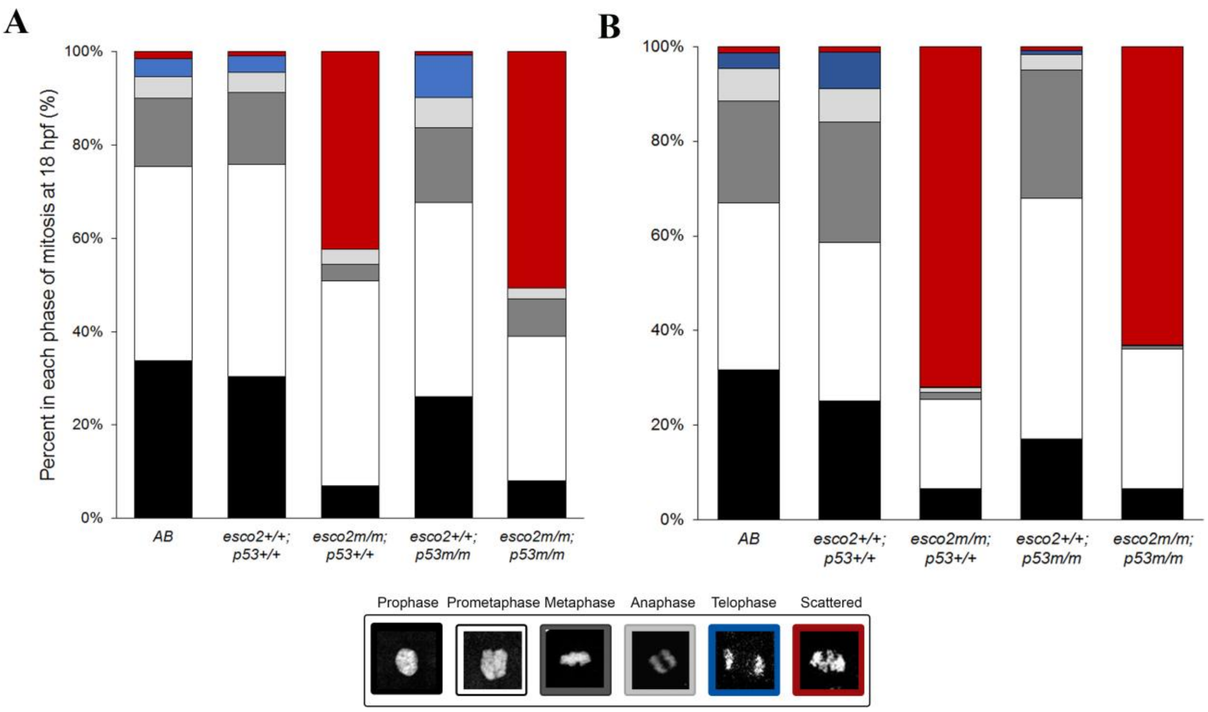

Supplemental Figure S4: Mitotic profiling in p53 mutant background has no effect on phase distribution at 18 and 30hpf. (A) Graph depicting the percentage of cells in each phase of mitosis between AB (the WT parental strain) controls, $esco2^{+/+}$, $esco2^{m/m}$, $esco2^{+/+}$; $p53^{m/m}$, and $esco2^{m/m}$; $p53m/m^{-}$ embryos at 18hpf (n=>70 morphologies/embryo, 3 embryos/genotype, mean ± st. dev.). **(B)** Graph depicting the percentage of cells in each phase of mitosis between AB (the WT parental strain) controls, $esco2^{+/+}$, $esco2^{m/m}$, $esco2^{+/+}$; $p53^{m/m}$, and $esco2^{m/m}$; $p53^{m/m}$ embryos at 30hpf (n=>70 morphologies/embryo, 3 embryos/genotype, mean ± st. dev.). Legend below demonstrates the pH3 morphology linked to the phase of mitosis used to quantitate these results.
